# Molecular Insights into SARS-CoV2-Induced Alterations of the Gut/Brain Axis

**DOI:** 10.3390/ijms221910440

**Published:** 2021-09-28

**Authors:** Serge Nataf, Laurent Pays

**Affiliations:** 1 INSERM, Stem Cell and Brain Research Institute U1208, Univ Lyon, Université Claude Bernard Lyon 1, 69500 Bron, France; laurent.pays@univ-lyon1.fr; 2 Bank of Tissues and Cells, Lyon University Hospital (Hospices Civils de Lyon), 69003 Lyon, France

**Keywords:** COVID-19, SARS-CoV2, enterocytes, long COVID, brain, trace amines, L-DOPA, monoamine oxidase

## Abstract

For a yet unknown reason, a substantial share of patients suffering from COVID-19 develop long-lasting neuropsychiatric symptoms ranging from cognitive deficits to mood disorders and/or an extreme fatigue. We previously reported that in non-neural cells, angiotensin-1 converting enzyme 2 (*ACE2*), the gene coding for the SARS-CoV2 host receptor, harbors tight co-expression links with dopa-decarboxylase (*DDC*), an enzyme involved in the metabolism of dopamine. Here, we mined and integrated data from distinct human expression atlases and found that, among a wide range of tissues and cells, enterocytes of the small intestine express the highest expression levels of *ACE2*, *DDC* and several key genes supporting the metabolism of neurotransmitters. Based on these results, we performed co-expression analyses on a recently published set of RNA-seq data obtained from SARS-CoV2-infected human intestinal organoids. We observed that in SARS-CoV2-infected enterocytes, *ACE2* co-regulates not only with *DDC* but also with a specific group of genes involved in (i) the dopamine/trace amines metabolic pathway, (ii) the absorption of microbiota-derived L-DOPA and (iii) the absorption of neutral amino acids serving as precursors to neurotransmitters. We conclude that in patients with long COVID, a chronic infection and inflammation of small intestine enterocytes might be indirectly responsible for prolonged brain alterations.

## 1. Introduction

The incidence of neurological manifestations in patients with COVID-19 is raising increasing concerns regarding the acute and long-term impacts of SARS-CoV2 on the central nervous system (CNS). In particular, in addition to anosmia and/or ageusia, possibly of peripheral origin, a large array of symptoms undoubtedly reflecting an alteration of brain functions were reported in patients infected by SARS-CoV2. Confusion, delirium and other hallmarks of a global encephalopathy are relatively frequent during the acute phase of the disease, notably in patients over 65 years of age and/or admitted in intensive care units [1,2,3]. However, during the so-called post-COVID phase, i.e., at distance from the acute infectious phase, other neuropsychiatric symptoms take center stage, such as memory loss, extreme fatigue, sleep disorders, anxiety, depression and psychotic symptoms [1,4,5,6]. In COVID or post-COVID patients exhibiting neurological and/or psychiatric symptoms, the imaging, functional and neuropathological investigations performed so far have led to conflicting results so that no clear and unequivocal etiology has gathered wide consensus yet. Indeed, current hypotheses fall into three main categories [7]: (i) the infection of neural cells by SARS-CoV2 [8,9,10], (ii) an autoimmune and/or neuroinflammatory process triggered at the periphery by the host anti-viral immune response [11,12,13] and (iii) an uncontrolled activation of the coagulation cascade leading to the generation of multiple brain microinfarcts [14,15,16]. Irrespective of the considered hypotheses, getting further insights into the pathophysiology of COVID-associated neuropsychiatric symptoms requires a deepening of our knowledge on the potential functions exerted by the human SARS-CoV2 receptor angiotensin-1 converting enzyme 2 (ACE2) on the control of cognition and behavior. In this context, we previously reported that, across a large number of microarray datasets exploring essentially non-neural tissues, dopa-decarboxylase (*DDC*) is the human gene exhibiting the closest co-expression link with *ACE2* [17]. It should be underscored that *DDC* not only is a key enzyme of the dopamine and serotonin synthetic pathways but also is necessary to the synthesis of trace amines [18], a group of monoamines comprising essentially the catecholamine precursor tyramine and the neuromediators tryptamine and beta-phenylethylamine (β-PEA). Interestingly also, ACE2 exhibits high expression levels in intestinal enterocytes [19,20,21] and, via its physical interaction with the neutral amino acid transporter SLC6A19 [22], supports the intestinal absorption of neutral amino acids that cross the blood–brain barrier and act as precursors for dopamine, serotonin and trace amines in the CNS. Importantly, it is well established that enteroendocrine cells of the intestine represent the main source of blood-circulating serotonin [23]. In contrast, only scattered data indicate that enterocytes, a non-endocrine cell type, actually express a full range of molecules supporting the metabolism of L-DOPA, dopamine and/or trace amines. Clarifying this point is all the more important for at least three reasons: (i) SARS-CoV2 was shown to acutely or chronically infect enterocytes in COVID-19 patients [24,25,26], (ii) ACE2 expressed by enterocytes exert mucosal immune functions that shape the composition of gut microbiota [27] and, potentially, the associated repertoire of microbiota-derived neuromediators [28], and (iii) patients with inflammatory bowel disease (IBD) exhibit both an intestinal down-regulation of ACE2 [29,30,31] and an abnormally high propensity to develop neuropsychiatric disorders [32,33].

In the present work, we first surveyed human expression atlases to extract results on the mRNA and protein levels exhibited by *DDC* and selected genes of the dopamine/trace amines synthetic pathways in enterocytes. In a second step, we then performed gene co-expression analyses on a recently published set of RNA-seq data obtained from SARS-CoV2-infected human intestinal organoids [34]. We found that *DDC* and key genes of the dopamine/trace amines synthetic pathways are not only highly expressed by enterocytes under normal conditions but also co-regulate with *ACE2* in SARS-CoV2-infected human intestinal organoids.

## 2. Results

### 2.1. Expression Patterns of ACE2, DDC and Key Genes of the Dopamine/Trace Amines Synthetic Pathways in Enterocytes of the Human Small Intestine

Assessment of the genomic consensus dataset of the Human Protein Atlas (HPA) (combining and integrating three large and independent datasets, as described in Materials and Methods) showed that, among 61 human tissues and cell types, the small intestine is the human tissue exhibiting the highest expression levels for *ACE2*, *SLC6A19*, *DDC*, *SLC7A9*, *MAOA* and *SULT1A2* (Table 1 and Supplementary Material S1).

The human small intestine was also in the top-5 tissues exhibiting the highest expression levels for *SLC3A1* (ranked N°2), *CYP2D6* (ranked N°2), *SULT1A1* (ranked N°5) and *SULT1A3* (ranked N°3) (Table 1 and Supplementary Material S1), all genes involved in the metabolism of dopamine and/or trace amines. In contrast, tyrosine hydroxylase (*TH*) mRNA levels were below the detection threshold in the human small intestine. To get insights into the identity of cells exhibiting such a pattern of expression in the human small intestine, we then mined the so-far largest single cell RNA-seq expression atlas currently available for human gut cells [35,36]. We observed that *ACE2* was included in the molecular signature of only two cell types and localizations: enterocytes of the small intestine and enteroendocrine cells of the small intestine (Table 2).

**Figure 1:**
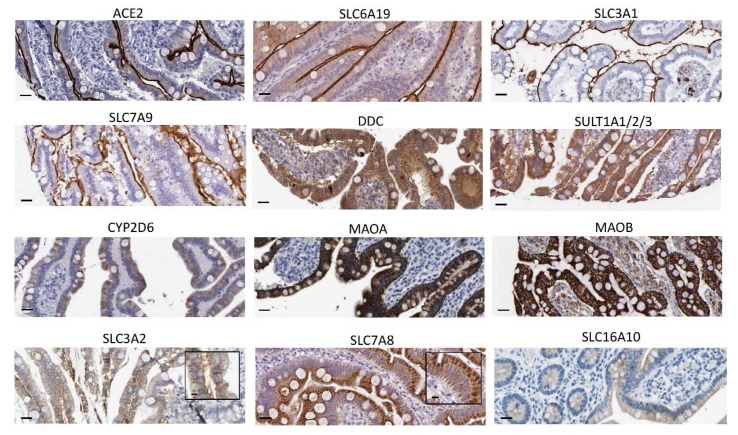
Expression by human enterocytes of key molecules involved in dopamine/trace amines metabolic pathways: A survey of the Human Protein Atlas (https://www.proteinatlas.org/ (accessed on 24 September 2021)) allowed extracting immunohistochemical data obtained on human small intestine for the following candidate molecules: angiotensin-converting enzyme 2 (ACE2), solute carrier family 6 member 19 (SLC6A19), solute carrier family 3 member 1 (SLC3A1), solute carrier family 7 member 9 (SLC7A9), dopa-decarboxylase (DDC), sulfotransferase family 1A member 1 (SULT1A1), sulfotransferase family 1A member 2 (SULT1A2), sulfotransferase family 1A member 3 (SULT1A3), cytochrome P450 family 2 subfamily D member 6 (CYP2D6), monoamine oxidase A (MAOA), monoamine oxidase B (MAOB), solute carrier family 3 member 2 (SLC3A2), solute carrier family 7 member 8 (SLC7A8) and solute carrier family 6 member 10 (SLC16A10). Scale bar: 25 μm.

Moreover, among the different cell types forming the small intestine epithelium (i.e., enterocytes, enteroendocrine cells, Paneth cells, goblet cells, intestinal stem cells or intestinal transit-amplifying cells), the molecular signature of enterocytes harbored the highest number of genes directly involved in the metabolism of dopamine and/or trace amines (nine genes). Other cell types expressing such genes of interest comprised Paneth cells (seven genes), goblet cells (five genes), enteroendocrine cells (four genes), stem cells (three genes) and transit-amplifying cells (two genes) (Table 2). Of note, none of the assessed genes of interest belonged to the molecular signature of colonic or rectal cells, whether these be enterocytes, enteroendocrine cells, Paneth cells, goblet cells, intestinal stem cells or intestinal transit-amplifying cells (Table 2). In contrast, irrespective of the cell type considered, the molecular signature of small intestine cells included genes involved in the metabolism of dopamine and/or trace amines. This observation suggests that regionalization rather than cell specificity may dictate the expression of such genes. At the protein level, a survey of the immunohistochemical analyses gathered in the Human Protein Atlas confirmed that enterocytes of the small intestine robustly express ACE2, SLC6A19 and the 12 other proteins we identified as molecules of interest due to their involvement in the metabolism of dopamine and/or trace amines (Figure 1). More details regarding antibodies and tissues are presented in Section 4.

**Figure 2:**
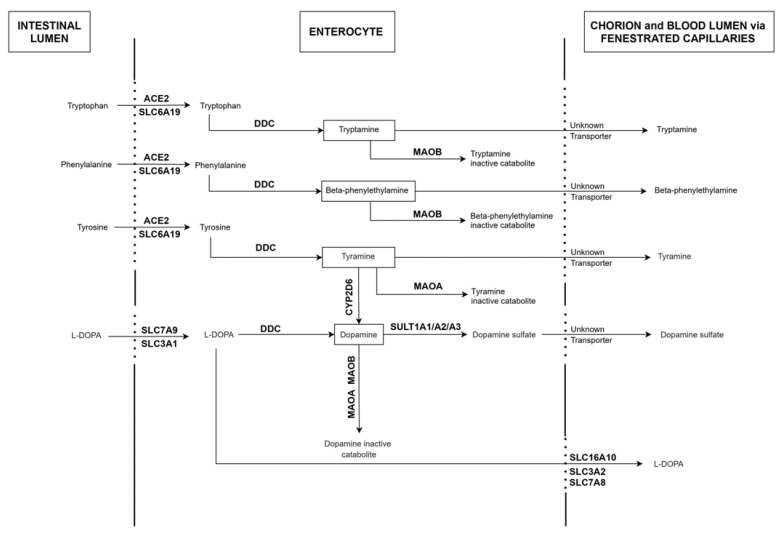
Functional scheme summarizing the predicted dopamine/trace amines metabolic pathways taking place in human enterocytes of the small intestine. This scheme is based on the mining of human expression atlases and on previously published biochemical and/or functional data obtained in intestinal or non-intestinal cells. The molecules included in this scheme comprise: angiotensin-converting enzyme 2 (ACE2), solute carrier family 6 member 19 (SLC6A19), solute carrier family 3 member 1 (SLC3A1), solute carrier family 7 member 9 (SLC7A9), dopa-decarboxylase (DDC), sulfotransferase family 1A member 1 (SULT1A1), sulfotransferase family 1A member 2 (SULT1A2), sulfotransferase family 1A member 3 (SULT1A3), cytochrome P450 family 2 subfamily D member 6 (CYP2D6), monoamine oxidase A (MAOA), monoamine oxidase B (MAOB), solute carrier family 3 member 2 (SLC3A2), solute carrier family 7 member 8 (SLC7A8) and solute carrier family 6 member 10 (SLC16A10).

It should be underscored that, as expected, ACE2 and SLC6A19, which mediate the influx transport of tryptophan, phenylalanine and tyrosine, both localized at the apical membrane of enterocytes. The same pattern of expression was observed for SLC3A1 and SLC7A9, which are involved in the influx transport of L-DOPA. In contrast, the enzymes DDC, SULT1A1/2/3, MAOA, MAOB and CYP2D6 harbored a cytoplasmic staining pattern. Additionally expected, the L-DOPA efflux transporters SLC3A2 and SLC7A8 were detected at the basolateral membrane of enterocytes. A low and diffuse staining pattern was observed for SLC16A10. Finally, no TH staining could be detected (Figure S1), in accordance with genomics analyses. Based on these mined data, a scheme summarizing the predicted dopamine/trace amines metabolic pathways taking place in human enterocytes is shown in Figure 2.

### 2.2. Assessment of Co-Expression Links between ACE2 and Key Genes of the Dopamine/Trace Amines Metabolic Pathways in SARS-CoV2-Infected Human Intestinal Organoids

We then sought to determine whether, in SARS-CoV2-infected human enterocytes, *ACE2* co-regulates with *DDC* and/or key genes involved in the dopamine/trace amines metabolic pathways. To this aim, we re-assessed a recently published RNA-seq dataset obtained from the analysis of control vs. SARS-CoV2-infected human intestinal organoids [34]. In these experiments, the expression of *ACE2* exhibited a peculiar kinetics characterized, at 24 h post-infection, by a dramatic drop of mRNA levels (by a factor > 10 in two independent experiments; Figure S2), followed by a return to baseline levels at 60 h post-infection (Figure S2). Among the genes of interest that we focused on, a similar silencing effect of SARS-CoV2 was observed at 24 h post-infection for *SLC6A19* (the gene encoding the neutral amino acid transporter that physically interacts with *ACE2* in enterocytes), *SLC7A9* (which codes for an L-DOPA influx transporter) and *SLC16A10* (which codes for an L-DOPA efflux transporter). From the whole set of data (*n* = 6, two control samples, two samples at 24 h post-infection and two samples at 60 h post infection), we could extract expression values for 11 out of 14 genes of interest. We then used the Pearson’s correlation test to evaluate the co-expression links between these genes and *ACE2*. We found that eight key genes involved in the metabolism of dopamine and/or trace amines exhibited statistically significant co-expression links with *ACE2* across all experimental conditions. Of note, the most robust correlation link was observed for *MAOB*, followed by *SLC7A9* and *SULT1A1* (Table 3).

Based on these correlation data, we could establish a list of candidate molecules with predictable altered blood levels in COVID-19 patients (Table 4).

**Figure 3:**
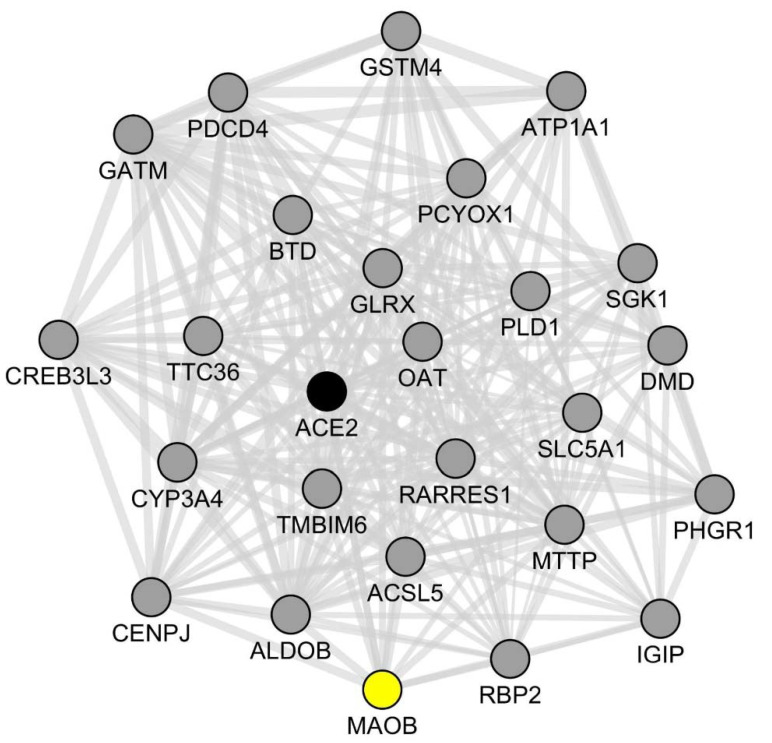
*ACE2* co-expression network in SARS-CoV2-infected human intestinal organoids. A set of previously published RNA-seq data [34] obtained from the analysis of SARS-CoV2-infected human intestinal organoids was re-assessed in order to identify the top-25 genes that more closely co-express with *ACE2* under these experimental conditions. Each gene is represented by its gene symbol and a plain circle. Each correlation link is represented by a plain gray line. The full list of *ACE2* co-expressed genes is shown in Table S1. *MAOB* (represented as a yellow plain circle), a crucial gene of the dopamine/trace amines metabolic pathways, co-expresses with *ACE2* (represented as a black plain circle) and other genes of the identified *ACE2* co-expression network.

To confirm and extend our results, we then used the GeneMANIA software to identify, in an unsupervised fashion, the top-25 genes exhibiting the most statistically significant co-expression links with *ACE2* in SARS-CoV2-infected intestinal organoids (Figure 3).

We found that *MAOB* belonged to this network along with two other genes involved in the metabolism of neutral amino-acids: *GATM* (glycine amidinotransferase) and *OAT* (ornithine aminotransferase). The full list of *ACE2*-co-expressed genes is shown in Table S1.

Further supporting a specific functional link between *ACE2* and *MAOB* in SARS-CoV2-infected human enterocytes, an enrichment analysis using the BioPlanet library of pathways (created and run by the National Institutes of Health, USA) [37], showed that the identified list of 25 *ACE2*-co-expressed genes comprised 7 *MAOB*-involving pathways among the top-10 most significantly enriched pathways (Table S2). These notably comprise the “arginine and proline metabolism” pathway (involving *MAOB*, *GATM* and *OAT*, adjusted *p*-value: 0.002), the “glycine, serine and threonine metabolism” pathway (involving *MAOB* and *GATM*, adjusted *p*-value: 0.011) and the “tryptophan metabolism” pathway (involving *MAOB* and *CYP3A4*, adjusted *p*-value: 0.029). It should be noticed that proline, glycine and serine are indeed neutral amino acids that are transported in enterocytes by the ACE2/SLC6A19 dimer.

## 3. Discussion

Our data mining of human expression atlases shows that key genes of the dopamine/trace amines synthetic pathways are highly expressed by human enterocytes of the small intestine. This observation indicates that enterocytes may participate in shaping the blood-circulating levels of the neuromediators L-DOPA, tryptamine and β-PEA, which are all endowed with the ability to cross the blood–brain barrier. In particular, the fact that intestinal enterocytes express high levels of the L-DOPA influx transporter SLC7A9, the L-DOPA-metabolizing enzyme DDC and the L-DOPA efflux transporters SLC16A10, SCL3A2 and SLC7A8 suggests that enterocytes shape the levels of blood-circulating L-DOPA. Our data mining observations are in line with previous studies demonstrating that, in human healthy subjects, blood-circulating L-DOPA essentially derives from the gastrointestinal tract and exhibits significant increased levels following food intake [38]. In this regard, one should keep in mind that besides food-derived L-DOPA [39,40,41,42], gut microbiota was firmly demonstrated to impact brain functions via the synthesis of L-DOPA [43]. Our observations also indicate that the physiological levels of blood-circulating tryptamine and β-PEA might be shaped by small intestine enterocytes. Indeed, enterocytes highly express three groups of molecules that are crucially involved in such a pathway: (i) ACE2 and SCL6A19, which allow the influx of L-tryptophan and phenylalanine from the intestinal lumen, (ii) DDC, which converts L-tryptophan and phenylalanine into tryptamine and β-PEA, respectively, and (iii) MAOB, which degrades tryptamine and β-PEA into inactive catabolites. Again, since tryptamine and β-PEA cross the blood–brain barrier [44,45,46,47] and exert neuromediator functions [48,49,50], our findings indicate that the enzymatic activity of DDC and MAOB in small intestine enterocytes may indirectly impact brain functions. Supporting this assumption, it was previously shown that in rats given an L-tryptophan-rich diet, the administration of an MAOA/MAOB inhibitor triggers a depressive-like behavior and a parallel increase in brain tryptamine levels [51]. Similarly, in human healthy subjects, the urinary excretion of tryptamine was found to increase by up to 7-fold following the oral administration of the MAOA/MAOB inhibitor tranylcypromine [52]. Additionally, *Maob*-deficient mice exhibit an abnormally high stress response, along with a nearly 10-fold increase in β-PEA contents in both brain and urine [53]. Finally, in patients suffering from severe depression, the administration of a MAOA/MAOB inhibitor along with an oral supplementation with L-phenylalanine was reported to exert beneficial effects via a mechanism presumably involving an increase in brain β-PEA levels [54]. It should be noticed that the trace amine tyramine, a catecholamine precursor that lacks neuromediator properties, is known to be actually metabolized in vivo by intestinal cells via the enzymatic activity of MAOA/MAOB. Indeed, when associated with the ingestion of tyramine-rich cheeses, the oral intake of MAOA/MAOB inhibitors is responsible for an adverse reaction named “cheese effect”, characterized by a rapid rise of blood-circulating tyramine and the subsequent development of a catecholamine-mediated hypertensive crisis [55,56]. Overall, since L-DOPA, tryptamine and β-PEA cross the blood–brain barrier, our findings point to a specific and underappreciated role of enterocytes in the control of mood and behavior.

The present study also provides evidence that key genes of the dopamine/trace amines synthetic pathways co-regulate with *ACE2* in SARS-CoV2-infected human enterocytes. In the study published by Lamers et al. [34], a drop in *ACE2* mRNA levels was observed in SARS-CoV2-infected human enterocytes at 24 h post-infection. This finding, which needs to be further replicated, is in line with previous reports obtained in SARS-CoV2-infected airway epithelial cells [57,58]. Authors from these studies and other researchers in the field proposed that the SARS-CoV2-induced dysregulation of *ACE2* plays a major role in COVID-19 pathophysiology. In this regard, readers should be reminded that SARS-CoV, a SARS-CoV2-related coronavirus responsible for the 2002–2004 SARS (severe acute respiratory syndrome) epidemics, was experimentally demonstrated to mediate respiratory symptoms via a down-regulation of *ACE2* in lung epithelial cells [59]. In any case, since human enterocytes express high levels of *ACE2* and are targeted by SARS-CoV2, genes identified as being co-regulated with *ACE2* in SARS-CoV2-infected enterocytes should be considered as potentially relevant in the context of COVID-19 pathophysiology. A supervised correlation analysis unraveled a close co-expression link between *ACE2* and *SLC6A19*, a transporter-coding gene whose protein product dimerizes with ACE2 and is indispensable for the intestinal absorption of neutral amino acids. The role of ACE2 and SLC6A19 in intestinal absorptive functions is well known. In particular, abnormally low levels of tryptophan are observed in the blood and brain of *Ace2* KO mice [27,60,61]. Interestingly, such alterations can be rescued by the oral administration of the dipeptide glycyl-tryptophan (Gly-Trp) [27], a tryptophan precursor whose absorption does not require the intestinal expression of ACE2. Additionally, human subjects bearing an *SLC6A19* loss-of-function mutation develop a disorder called Hartnup disease, characterized notably by the co-occurrence of neuropsychiatric symptoms and low blood levels of neutral amino acids [62,63]. In addition to *SLC6A19*, we also found that in SARS-CoV2-infected enterocytes, *ACE2* co-regulates with several crucial genes of the dopamine/trace amines synthetic pathways. The most statistically significant correlation links with *ACE2* were observed for *SLC7A9* and *MAOB*, two genes respectively involved in the intestinal absorption of L-DOPA and the catabolism of both tryptamine and β-PEA. As a matter of fact, dopamine and trace amines have been involved in the pathophysiology of bipolar disorder [64,65,66] and schizophrenia [67,68,69,70].

We also performed an unsupervised correlation analysis allowing identification of the top-25 genes exhibiting the closest correlation links with *ACE2* in SARS-CoV2-infected enterocytes. *MAOB* belonged to this short list of genes, reinforcing the notion that in human enterocytes, SARS-CoV2-induced dysregulation of *ACE2* might be accompanied by alterations of the dopamine/trace amines metabolic pathways. Only a few system biology metabolomics analyses have been performed so far on blood samples from COVID-19 patients. Strikingly, one such study showed that among the metabolites exhibiting altered blood levels in COVID-19 patients (*n* = 33), the highest scores of statistical enrichment were observed for metabolites related to neutral amino acids metabolic pathways (notably tryptophan, proline and/or lysine metabolic pathways) (Figure S3A) [71]. In this dataset, neutral amino acids such as tryptophan, alanine, glycine, serine and glutamine were found to be down-regulated in the blood of COVID-19 patients, irrespective of the presence of high or moderate levels of systemic inflammation (Figure S3B,C). Raw data regarding the blood levels of tyrosine are unfortunately not accessible in this report. However, another metabolomics study [72] in which a larger cohort of patients was analyzed (*n* = 50) clearly demonstrated decreased blood levels of neutral amino acids (including tryptophan and tyrosine) in either severe or non-severe COVID-19 patients when compared with healthy subjects (Table S3). Interestingly also, this study reported a statistically significant decrease in dopamine sulfate (dopamine 3-O-sulfate) in the blood of non-severe COVID-19 patients as compared with healthy controls (Table S3). Of note, measuring trace amines in biological fluids requires techniques that are more sensitive than mass spectrometry. Similarly, the blood of healthy subjects contains high amounts of dopamine-sulfate but only low levels of L-DOPA (which nonetheless increase following food intake) [38]. These technical issues may explain the current lack of data regarding such molecules in the context of COVID-19.

Overall, since SARS-CoV2 was shown to chronically infect human enterocytes in vivo, our findings raise the possibility that in patients with long COVID, a dysfunction of ACE2 in human enterocytes might alter the blood levels of molecules that shape or impact brain functions. These notably include neutral amino acids L-DOPA, tryptamine and β-PEA.

## 4. Materials and Methods

### 4.1. Mining of Human Expression Atlases

Data mining analyses were performed and repeated at least 3 times between September 2020 and July 2021. We limited our survey of human expression atlases to key genes allowing the conversion of tyrosine, tryptophan and/or phenylalanine into dopamine and/or trace amines. We also included in our search the sulfatases SULT1A1, SULT1A2 and SULT1A3, which support the conversion of dopamine into dopamine-sulfate, the main blood-circulating form of dopamine [38,73]. Finally, we took into consideration the fact that L-DOPA is physiologically synthesized by gut microbiota [28,74] and is absorbed by intestinal enterocytes via specific influx transporters (SLC7A9 and SLC3A1) and efflux transporters (SLC16A10, SLC7A8 and SLC3A2) [75]. On this basis, the following genes of interest were thus retained for further analyses:

∗ Dopa-decarboxylase (*DDC*): an enzyme allowing the synthesis of dopamine from L-DOPA, the synthesis of tyramine from tyrosine [43], the synthesis of beta-phenylethylamine (β-PEA) from phenylalanine and the synthesis of tryptamine from tryptophane [18,76];
∗ Cytochrome P450 family 2 subfamily D member 6 (*CYP2D6*): an enzyme allowing the synthesis of dopamine from tyramine [43];
∗ Solute carrier family 7 member 9 (*SLC7A9*) and solute carrier family 3 member 1 (*SLC3A1*): transporters allowing the cellular influx of L-DOPA [75];
∗ Solute carrier family 6 member 10 (*SLC16A10*), solute carrier family 7 member 8 (*SLC7A8*) and solute carrier family 3 member 2 (*SLC3A2*): transporters allowing the cellular efflux of L-DOPA [75];
∗ Sulfotransferase family 1A member 1 (*SULT1A1*), sulfotransferase family 1A member 2 (*SULT1A2*), sulfotransferase family 1A member 3 (*SULT1A3*): enzymes allowing the sulfation of dopamine, leading to the generation of dopamine 3-O-sulfate and dopamine 4-O-sulfate [38,73];
∗ Monoamine oxidase A (*MAOA*): an enzyme essentially allowing the catabolism of dopamine and tyramine [77,78];
∗ Monoamine oxidase B (*MAOB*): an enzyme allowing the catabolism of dopamine, tryptamine and β-PEA [53,78,79].

In parallel, the same transcriptomic datasets were mined to assess in human small intestine enterocytes the basal expression of *ACE2* (angiotensin-converting enzyme 2) and *SLC6A19* (solute carrier family 6 member 19, the gene coding for the neutral amino acid transporter that physically interacts with ACE2). As a negative control, we also extracted data regarding the enterocytic expression of tyrosine hydroxylase (*TH*), a gene supporting the generation of L-DOPA from tyrosine and whose expression pattern is limited to catecholaminergic cells of the adrenal glands and to dopaminergic neurons [80]. To explore in human small intestine enterocytes the steady state expression levels of the above-mentioned set of genes, we first extracted results gathered in the genomics and proteomics database “Human Protein Atlas” (HPA) (https://www.proteinatlas.org/ (accessed on 24 September 2021)) [81]. In particular, we queried the HPA consensus dataset, which was obtained by compiling and normalizing the currently 3 largest mRNA expression atlases obtained from the analysis of normal human tissues and cells: the HPA dataset (https://www.proteinatlas.org/ (accessed on 24 September 2021)), the FANTOM5 dataset (https://fantom.gsc.riken.jp/5/ (accessed on 24 September 2021)) [82] and the GTEx dataset (https://gtexportal.org/home/ (accessed on 24 September 2021)) [83]. The bioinformatics method used to combine and normalize data from these 3 distinct sources is described in detail on the HPA website (https://www.proteinatlas.org/ (accessed on 24 September 2021)). In brief, for each of the 3 transcriptomics datasets (HPA, GTEx and FANTOM5), the average transcripts per kilobase million (TPM) value of all individual samples for each human tissue or human cell type was extracted. All TPM values of all the samples within each data source were normalized using the trimmed mean of M values (TMM) method, followed by Pareto scaling of each gene within each data source. Tissue data from the three transcriptomics datasets were subsequently integrated using batch correction through the “removeBatchEffect” function of R package Limma, using the data source as a batch parameter. The resulting transcript expression values, denoted normalized expression (NX), were then calculated for each gene in every sample. Mining the HPA consensus dataset for each queried gene thus allowed us to rank mRNA levels in the human small intestine as compared with 60 other human tissues. As a confirmatory investigation, we explored a recently published expression atlas of the human intestine obtained by single-cell RNA-seq analyses of human gut cells [35,36]. Finally, to obtain insights into expression patterns at the protein level, we mined histological data in the Human Protein Atlas and, for each protein of interest, extracted results obtained by immunohistochemistry on sections of normal human small intestine. Below are listed URLs where the references of antibodies and a detailed description of each tissue staining can be found:

ACE2: https://www.proteinatlas.org/ENSG00000130234-ACE2/tissue/small+intestine (accessed on 24 September 2021); SLC6A19: https://www.proteinatlas.org/ENSG00000174358-SLC6A19/tissue/small+intestine (accessed on 24 September 2021); SLC7A9: https://www.proteinatlas.org/ENSG00000021488-SLC7A9/tissue/small+intestine (accessed on 24 September 2021); SLC3A1: https://www.proteinatlas.org/ENSG00000138079-SLC3A1/tissue/small+intestine (accessed on 24 September 2021); SLC3A2: https://www.proteinatlas.org/ENSG00000168003-SLC3A2/tissue/small+intestine (accessed on 24 September 2021); SLC7A8: https://www.proteinatlas.org/ENSG00000092068-SLC7A8/tissue/small+intestine (accessed on 24 September 2021); SLC16A10: https://www.proteinatlas.org/ENSG00000112394-SLC16A10/tissue/small+intestine (accessed on 24 September 2021); *DDC*: https://www.proteinatlas.org/ENSG00000132437-DDC/tissue/small+intestine (accessed on 24 September 2021); MAOA: https://www.proteinatlas.org/ENSG00000189221-MAOA/tissue/small+intestine (accessed on 24 September 2021); MAOB: https://www.proteinatlas.org/ENSG00000069535-MAOB/tissue/small+intestine (accessed on 24 September 2021); CYP2D6: https://www.proteinatlas.org/ENSG00000100197-CYP2D6/tissue/small+intestine (accessed on 24 September 2021); SULT1A1: https://www.proteinatlas.org/ENSG00000196502-SULT1A1/tissue/small+intestine (accessed on 24 September 2021); SULT1A2: https://www.proteinatlas.org/ENSG00000197165-SULT1A2/tissue/small+intestine (accessed on 24 September 2021); SULT1A3: https://www.proteinatlas.org/ENSG00000261052-SULT1A3/tissue/small+intestine (accessed on 24 September 2021); TH: https://www.proteinatlas.org/ENSG00000180176-TH/tissue/small+intestine (accessed on 24 September 2021).

### 4.2. Gene Co-Expression Analyses

To evaluate the impact of SARS-CoV2 on the intestinal expression of *ACE2*, *DDC* and key genes of the dopamine/trace amines synthetic pathways, we re-assessed a recently published dataset in which RNA-seq analyses were performed on control vs. SARS-CoV2-infected human intestinal organoids [34]. We extracted data that had been obtained under 3 experimental conditions: differentiated human intestinal organoids in control conditions (*n* = 2), differentiated human intestinal organoids at 24 h following infection with SARS-CoV2 (*n* = 2), differentiated human intestinal organoids at 60 h following infection with SARS-CoV2 (*n* = 2). We then assessed across samples from these distinct experimental conditions the co-expression of *ACE2* with *DDC* and with key genes involved in the metabolism of dopamine and/or trace amines. Two analytical approaches were followed concurrently: (i) the calculation of Pearson’s correlation coefficients between *ACE2* and genes of interest, (ii) the unsupervised identification of the 25 genes being the most closely co-expressed with *ACE2* among a total of 18,011 genes with reported values. To this aim, we used the network visualization software Cytoscape [84] and the gene co-expression plugin GeneMANIA [85], as previously described [86].

## 5. Conclusions

Altogether our observations indicate that the chronic infection of intestinal enterocytes by SARS-CoV2 might be indirectly responsible for the neuropsychiatric symptoms reported in patients with long COVID. A clinical support to this view is provided by a recent work showing that the occurrence of gastrointestinal symptoms during the acute phase of the disease is a clinical predictor of cognitive alterations during the so-called post-COVID phase [87]. We suggest that future investigations performed in patients with COVID-19-associated neuropsychiatric symptoms should include (i) measures of blood-circulating neutral amino acids L-DOPA, tryptamine and β-PEA and (ii) endoscopic intestinal biopsies in order to assess the persistence of SARS-CoV2 in enterocytes, the expression levels of ACE2 and the existence of a local low-grade chronic inflammation. Finally, our work supports the biological relevance of therapeutic strategies based on the enteral and/or parenteral supplementation in neutral amino acids.

## Figures and Tables

**Table 1:** Ranks of mRNA expression levels reported in the human small intestine for *ACE2*, *SLC6A19* and key genes of the dopamine/trace amine metabolic pathways.

Gene Symbol	Rank Reported for the Small Intestine among 61 Human Tissues
*ACE2*	1
*SLC6A19*	1
*SLC7A9*	1
*SLC3A1*	2
*SLC3A2*	20
*SLC7A8*	30
*SLC16A10*	13
*DDC*	1
*MAOA*	1
*MAOB*	14
*CYP2D6*	2
*SULT1A1*	5
*SULT1A2*	1
*SULT1A3*	3
*TH*	not detected

Among 61 human tissues and cells for which mRNA expression values are compiled in the HPA consensus dataset, the small intestine ranks in the top-5 of the localizations exhibiting the highest mRNA levels for *ACE2*, *DDC* and several key genes of the dopamine/trace amines metabolic pathways. Gene symbols: angiotensin-converting enzyme 2 (*ACE2*), solute carrier family 6 member 19 (*SLC6A19*)*,* solute carrier family 7 member 9 (*SLC7A9*), solute carrier family 3 member 1 (*SLC3A1*)*,* solute carrier family 3 member 2 (*SLC3A2*), solute carrier family 7 member 8 (*SLC7A8*), solute carrier family 16 member 10 (*SLC16A10*), dopa-decarboxylase (*DDC*), monoamine oxidase A (*MAOA*), monoamine oxidase B (*MAOB*), cytochrome P450 family 2 subfamily D member 6 (*CYP2D6*), sulfotransferase family 1A member 1 (*SULT1A1*), sulfotransferase family 1A member 2 (*SULT1A2*)*,* sulfotransferase family 1A member 3 (*SULT1A3*).

**Table 2:** Mining of single cell RNA-seq data obtained from the analysis of human gut cells.

Cell Type and Intestinal Segment	Genes of Interest with Reported Presence in the Molecular Signatures
**Enterocytes**	
ileum	*ACE2, SLC6A19, SLC7A9, SLC3A1, DDC, MAOA, MAOB, CYP2D6, SULT1A1, SULT1A2, SULT1A3*
colon rectum	none none
**Enteroendocrine cells** ileum colon rectum	*ACE2, SLC6A19, SLC7A9, SLC3A1, MAOA, SULT1A2* none none
**Paneth cells**	
ileum	*SLC6A19, SLC7A9, SLC3A1, DDC, MAOA, CYP2D6, SULT1A1, SULT1A2*
colon rectum	none none
**Goblet cells** ileum colon rectum	*SLC7A9, DDC, MAOA, SULT1A1, SULT1A2* none none
**Stem cells** ileum colon rectum	*DDC, MAOA, SLC3A1,* none none
**Transit amplifying cells** illeum colon rectum	*DDC, MAOA* none none

Gene symbols: angiotensin-converting enzyme 2 (*ACE2*), solute carrier family 6 member 19 (*SLC6A19*), solute carrier family 7 member 9 (*SLC7A9*), solute carrier family 3 member 1 (*SLC3A1*), dopa-decarboxylase (*DDC*), monoamine oxidase A (*MAOA*), monoamine oxidase B (*MAOB*), cytochrome P450 family 2 subfamily D member 6 (*CYP2D6*), sulfotransferase family 1A member 1 (*SULT1A1*), sulfotransferase family 1A member 2 (*SULT1A2*)*,* sulfotransferase family 1A member 3 (*SULT1A3*).

**Table 3:** Correlation analysis of ACE2 mRNA levels with key genes of the dopamine/trace amines metabolic pathways in SARS-CoV2-infected human enterocytes.

*DDC*	*MAOA*	*MAOB*	*SULT1A1*	*SLC7A9*	*SLC3A1*	*SLC6A19*	*SLC3A2*
0.84	0.86	0.96	0.92	0.95	0.87	0.88	0.9
0.035	0.025	0.001	0.007	0.003	0.02	0.017	0.013

Expression data were extracted from Lamers et al. [34] and the Pearson’s test was applied to assess correlation coefficient (r, upper line) and statistical significance (*p*-value, lower line)) between ACE2 and genes of interest. Gene symbols: dopa-decarboxylase (*DDC*), monoamine oxidase A (*MAOA*), monoamine oxidase B (*MAOB*), solute carrier family 7 member 9 (*SLC7A9*), solute carrier family 3 member 1 (*SLC3A1*), solute carrier family 6 member 19 (*SLC6A19*), solute carrier family 3 member 2 (*SLC3A2*).

**Table 4:** Metabolites and neuromediators with predictable altered blood levels in patients with COVID-19.

Molecular Processes Putatively Targeted by SARS-CoV2 in Human Enterocytes	Molecules with Predictable Altered Blood Levels
Influx of L-DOPA (*SLC3A1**,* *SLC7A9*) Efflux of L-DOPA (*SLC16A10*, *SLC3A2*, *SLC7A8*) Conversion of L-DOPA into Dopamine (*DDC*) Catabolism of Dopamine (*MAO-A**,* *MAO-B*) Sulfation of Dopamine (*SULT1A1**, SULT1A2, SULT1A3*)	**L-DOPA** Dopamine Dopamine sulfate
Influx of Tyrosine (*ACE2*, *SLC6A19*) Conversion of Tyrosine into tyramine (*DDC*) Catabolism of Tyramine (*MAO-A*)	**Tyramine**, Tyrosine Dopamine Dopamine sulfate
Influx of Phenylalanine (*ACE2*, *SLC6A19*) Conversion of Phenylalanine into β-PEA (*DDC*) Catabolism of β-PEA (*MAO-B*)	**β-PEA**, Phenylalanine
Influx of Tryptophan (*ACE2*, *SLC6A19*) Conversion of Tryptophan into Tryptamine (*DDC*) Catabolism of Tryptamine (*MAO-B*)	**Tryptamine**, Tryptophan

Based on the demonstrated correlation links between *ACE2* and genes of interest, metabolite and/or neuromediators with predictable altered blood levels in COVID-19 patients were identified. Genes that co-express with *ACE2* in SARS-CoV2-infected human intestinal organoids are highlighted in red. Molecules exerting neuromediator functions and crossing the blood–brain barrier are indicated in bold letters. Gene symbols: dopa-decarboxylase (*DDC*), monoamine oxidase A (*MAOA*), monoamine oxidase B (*MAOB*), solute carrier family 7 member 9 (*SLC7A9*), solute carrier family 3 member 1 (*SLC3A1*), solute carrier family 6 member 19 (*SLC6A19*), angiotensin-converting enzyme 2 (*ACE2*), dopa-decarboxylase (*DDC*), monoamine oxidase A (*MAOA*), monoamine oxidase B (*MAOB*), sulfotransferase family 1A member 1 (*SULT1A1*), sulfotransferase family 1A member 2 (*SULT1A2*)*,* sulfotransferase family 1A member 3 (*SULT1A3*).

## Data Availability

All the data analyzed in this study are publically available and can be found by consulting the corresponding references (web sites or articles) listed in Section 4 of the present paper.

## Supplementary Materials

Data supplements are available online at https://www.mdpi.com/article/10.3390/ijms221910440/s1.
