# Supplementary material for: Molecular Insights into SARS-CoV2-Induced Alterations of the Gut/Brain Axis
Source: Int J Mol Sci. 2021 Sep 28;22(19):10440. doi: 10.3390/ijms221910440 (PMC8508788; doi:10.3390/ijms221910440)

Figure S1: Immunohistological assessment of Tyrosine Hydroxylase (TH) expression by intestinal human enterocytes. A survey of the Human Protein Atlas (https://www.proteinatlas.org/) allowed extracting immunohistochemical data obtained on human small intestine for the molecule Tyrosine Hydroxylase (*TH*). Scale bar: 25 μm.

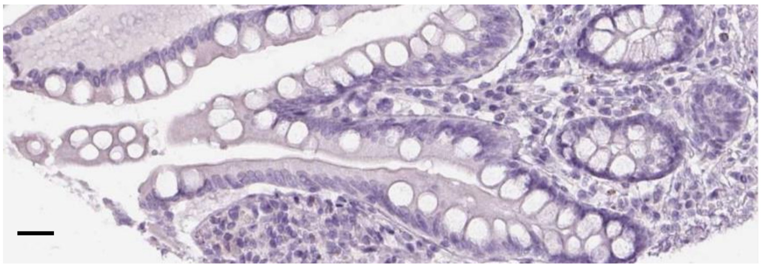

Supplement: Supplementary file 1 [file ijms-22-10440-s001.zip › Figure S1.pdf]
