# Supplementary material for: Molecular Insights into SARS-CoV2-Induced Alterations of the Gut/Brain Axis
Source: Int J Mol Sci. 2021 Sep 28;22(19):10440. doi: 10.3390/ijms221910440 (PMC8508788; doi:10.3390/ijms221910440)
Supplement: Supplementary file 1 [file ijms-22-10440-s001.zip › Figure S2.pdf]

Figure S2: Kinetics of *ACE2*, *SLC6A19*, *SLC7A9* and *SLC16A10* mRNA levels in SARS-CoV2-infected human enterocytes. Data were extracted from a recently published dataset in which RNA-seq analyses were performed on control vs SARS-CoV2 infected human intestinal organoids [34]. RNA-seq values expressed as normalized reads per million (RPM) are shown for *ACE2*, *SLC6A19*, *SLC7A9* and *SLC16A10* under 3 experimental conditions: differentiated human intestinal organoids in control conditions (n = 2), differentiated human intestinal organoids at 24h following infection with SARS-Cov2 (n = 2), differentiated human intestinal organoids at 60h following infection with SARS-Cov2 (n = 2).

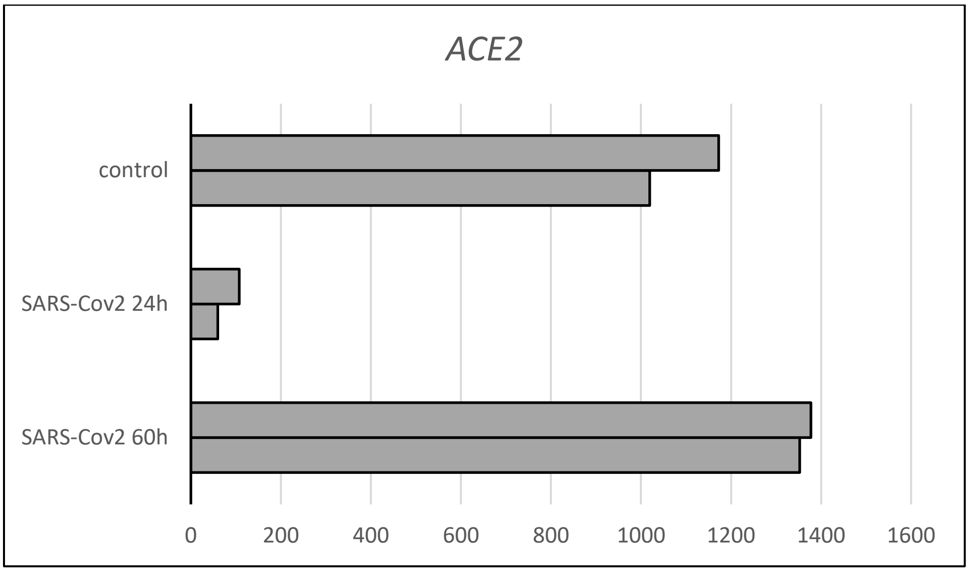

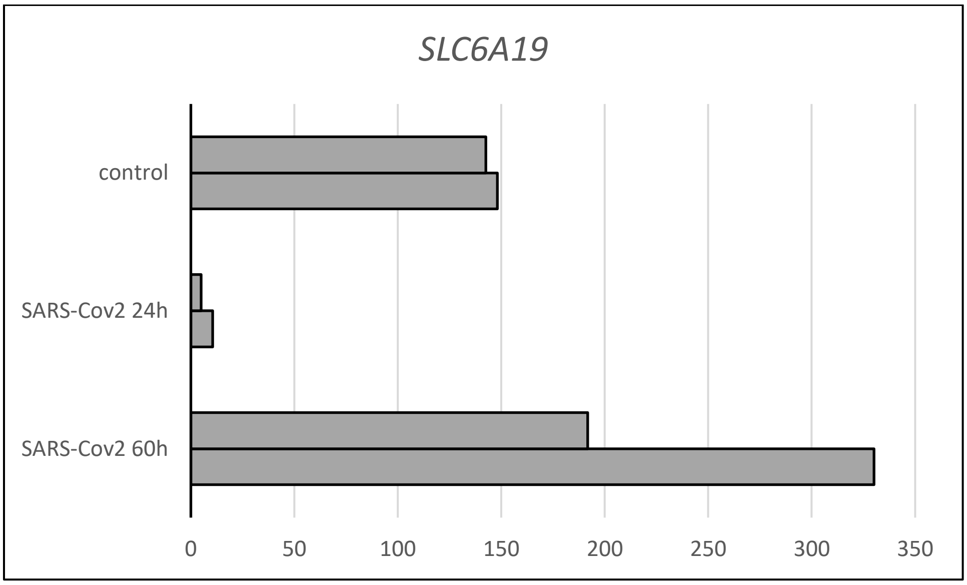

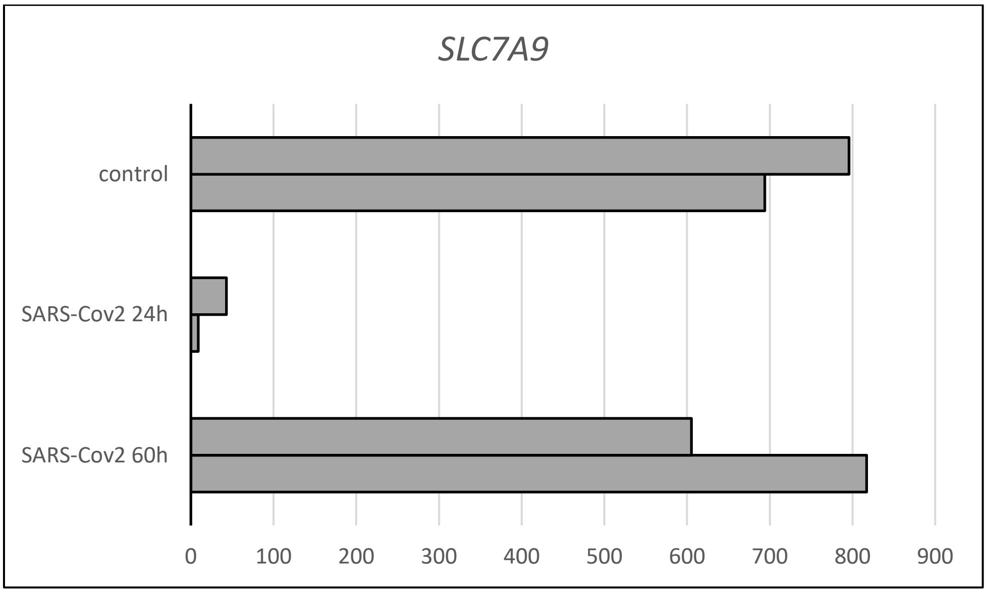
SLC7A9
control
SARS-Cov2 24h
SARS-Cov2 60h
0
100
200
300
400
500
600
700
800
900

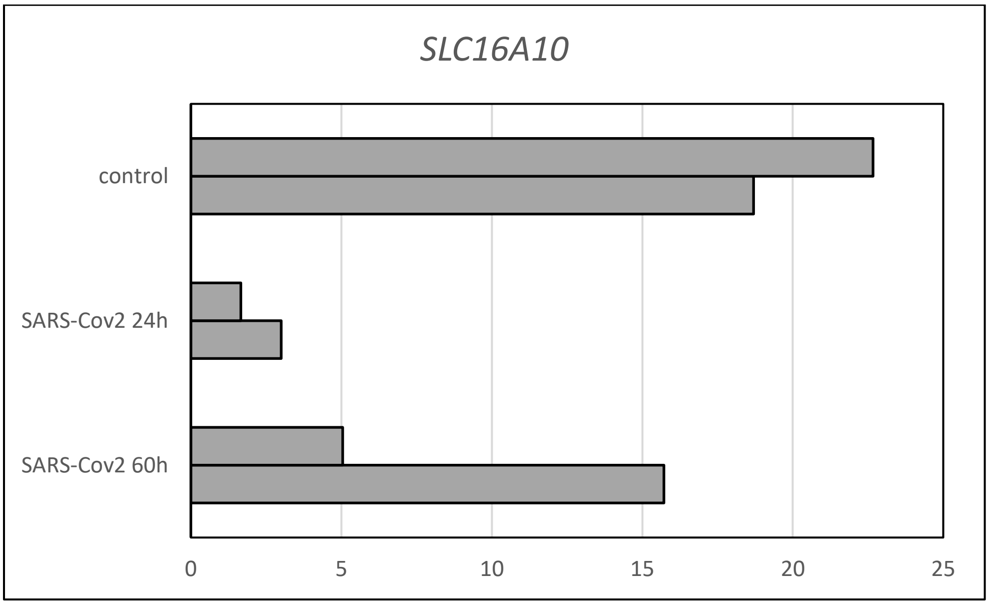
SLC16A10
control
SARS-Cov2 24h
SARS-Cov2 60h
0
5
10
15
20
25
