# Supplementary material for: Molecular Insights into SARS-CoV2-Induced Alterations of the Gut/Brain Axis
Source: Int J Mol Sci. 2021 Sep 28;22(19):10440. doi: 10.3390/ijms221910440 (PMC8508788; doi:10.3390/ijms221910440)

**Figure S3: Results extracted from metabolomics analyses performed on blood samples from COVID-19 patients.** Relevant results from two recently-published metabolomics studies [71, 72] are presented below in E1-E3 (results extracted from Ref [71]) and E4 (results extracted from Ref [72]).

**A:** Enrichment analysis from the whole list of metabolites exhibiting altered blood levels in COVID-19 patients as compared to controls [71].

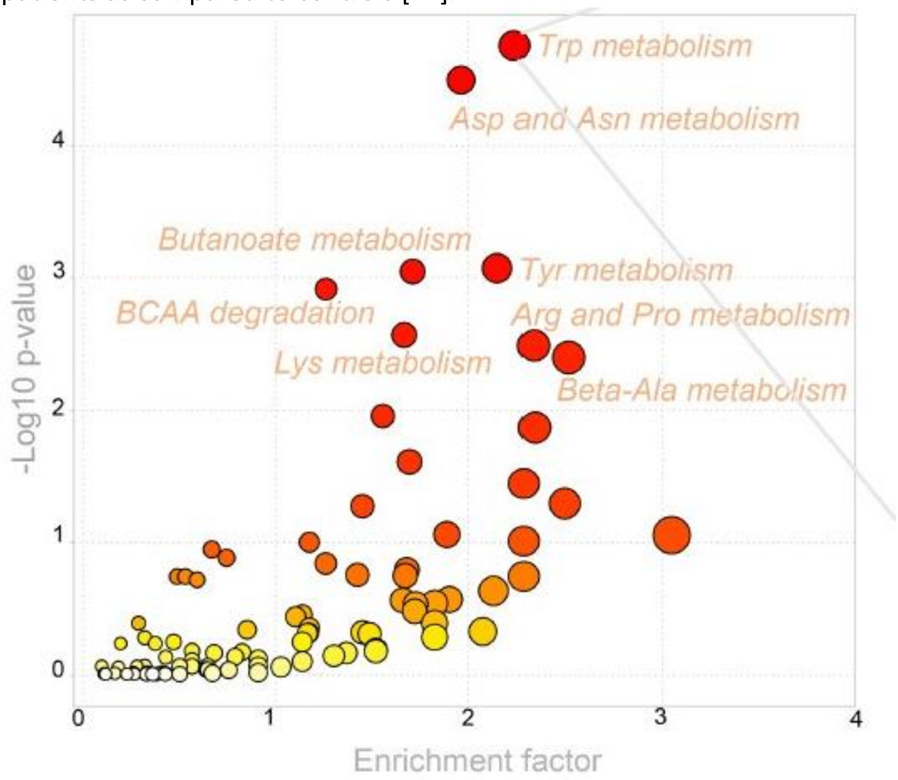

**B:** Measures of tryptophan levels (expressed in μM) in the blood of COVID-19 patients as compared to controls [71].

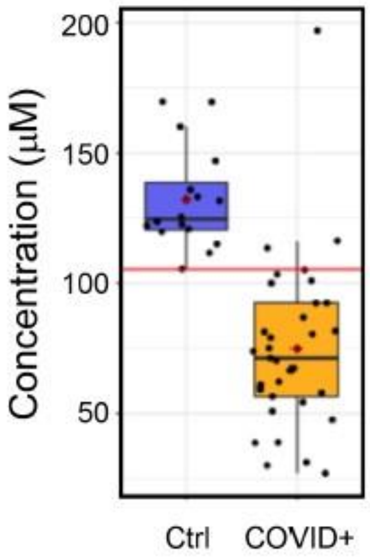

**C:** Measures of neutral amino acids (expressed in arbitrary units) as assessed by mass spectrometry in the blood of controls patients (grey circles) vs COVID patients with different blood levels of interleukine-6 (IL-6)(light blue circles = low IL-6 levels ; dark blue circle = medium IL-6 levels ; purple circles = high IL-6 levels) [71].

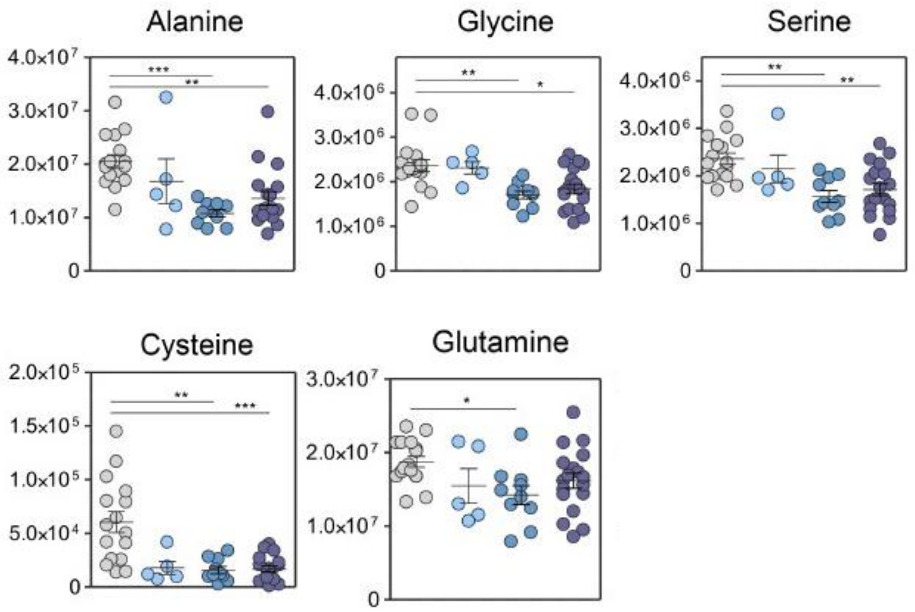

Supplement: Supplementary file 1 [file ijms-22-10440-s001.zip › Figure S3.pdf]
