# Supplementary material for: Molecular Insights into SARS-CoV2-Induced Alterations of the Gut/Brain Axis
Source: Int J Mol Sci. 2021 Sep 28;22(19):10440. doi: 10.3390/ijms221910440 (PMC8508788; doi:10.3390/ijms221910440)
Supplement: Supplementary file 1 [file ijms-22-10440-s001.zip › Supplementary material S1.pdf]

To explore in human small intestine enterocytes the steady state mRNA expression levels of genes of interest, we extracted results gathered in the genomics database “Human Protein Atlas” (HPA) (https://www.proteinatlas.org/) [82]. More specifically, we queried the HPA consensus dataset which was obtained by compiling and normalizing the currently 3 largest mRNA expression atlases obtained from the analysis of normal human tissues and cells: the HPA dataset (https://www.proteinatlas.org/), the FANTOM5 dataset (https://fantom.gsc.riken.jp/5/) [83] and the GTEx dataset (https://gtexportal.org/home/) [84]. The bioinformatics method used to combine and normalize data from these 3 distinct sources is described in details on the HPA website (https://www.proteinatlas.org/). In brief, for each of the 3 transcriptomics datasets (HPA, GTEx and FANTOM5), the average Transcripts Per Kilobase Million (TPM) value of all individual samples for each human tissue or human cell type was extracted. All TPM values of all the samples within each data source were normalized using the trimmed mean of M values (TMM) method, followed by Pareto scaling of each gene within each data source. Tissue data from the three transcriptomics datasets were subsequently integrated using batch correction through the “removeBatchEffect” function of R package Limma, using the data source as a batch parameter. The resulting transcript expression values, denoted Normalized eXpression (NX), were then calculated for each gene in every sample.

Below are shown the Normalized eXpression values (NX) reported in the HPA genomics atlas in 61 human tissues for the genes of interest we selected. Genes exhibiting the highest rank of expression in the small intestine are first shown.

ACE2

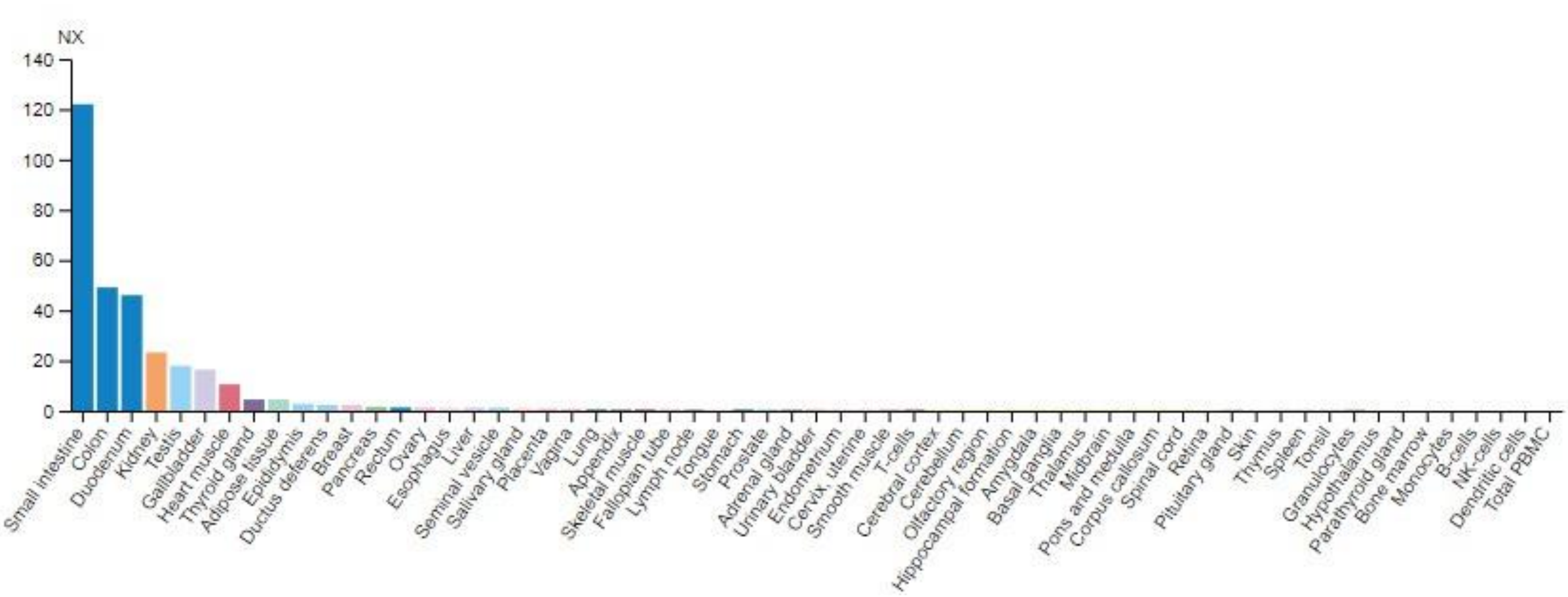
NX
140
120
100
80
60
40
20
0
Small intestine
Colon
Duodenum
Kidney
Testis
Gallbladder
Heart muscle
Thyroid gland
Adipose tissue
Epididymis
Ductus deferens
Breast
Pancreas
Rectum
Ovary
Esophagus
Liver
Seminal vesicle
Salivary gland
Placenta
Vagina
Lung
Appendix
Skeletal muscle
Fallopian tube
Lymph node
Tongue
Stomach
Prostate
Adrenal gland
Urinary bladder
Endometrium
Cervix, uterine
Smooth muscle
T-cells
Cerebral cortex
Cerebellum
Olfactory region
Hippocampal formation
Amygdala
Basal ganglia
Thalamus
Midbrain
Pons and medulla
Corpus callosum
Spinal cord
Retina
Pituitary gland
Skin
Thymus
Spleen
Tonsil
Granulocytes
Hypothalamus
Parathyroid gland
Bone marrow
Monocytes
B-cells
NK-cells
Dendritic cells
Total PBMC

DDC

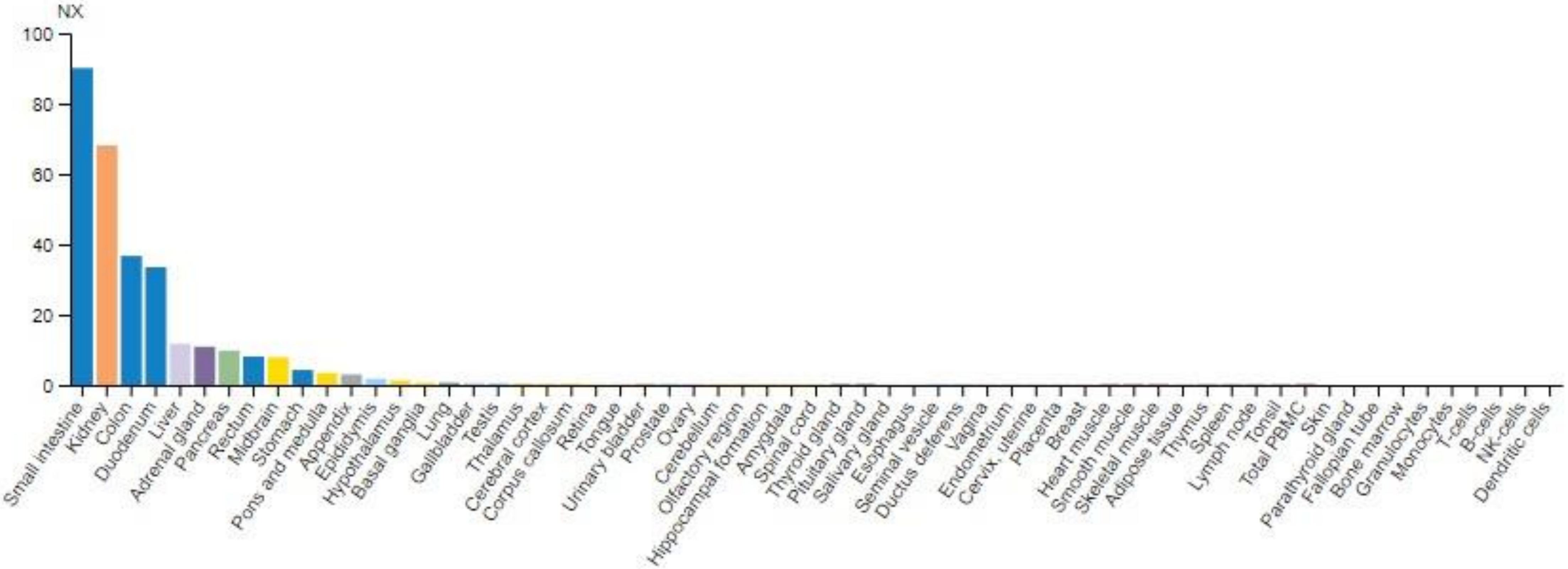
NX
100
80
60
40
20
0
Small intestine
Kidney
Colon
Duodenum
Liver
Adrenal gland
Pancreas
Rectum
Midbrain
Stomach
Pons and medulla
Appendix
Epididymis
Hypothalamus
Basal ganglia
Lung
Gallbladder
Testis
Thalamus
Cerebral cortex
Corpus callosum
Retina
Tongue
Urinary bladder
Prostate
Ovary
Cerebellum
Olfactory region
Hippocampal formation
Amygdala
Spinal cord
Thyroid gland
Pituitary gland
Salivary gland
Esophagus
Seminal vesicle
Ductus deferens
Vagina
Endometrium
Cervix, uterine
Placenta
Breast
Heart muscle
Smooth muscle
Skeletal muscle
Adipose tissue
Thymus
Spleen
Lymph node
Tonsil
Total PBMC
Skin
Parathyroid gland
Fallopian tube
Bone marrow
Granulocytes
Monocytes
T-cells
B-cells
NK-cells
Dendritic cells

MAOA

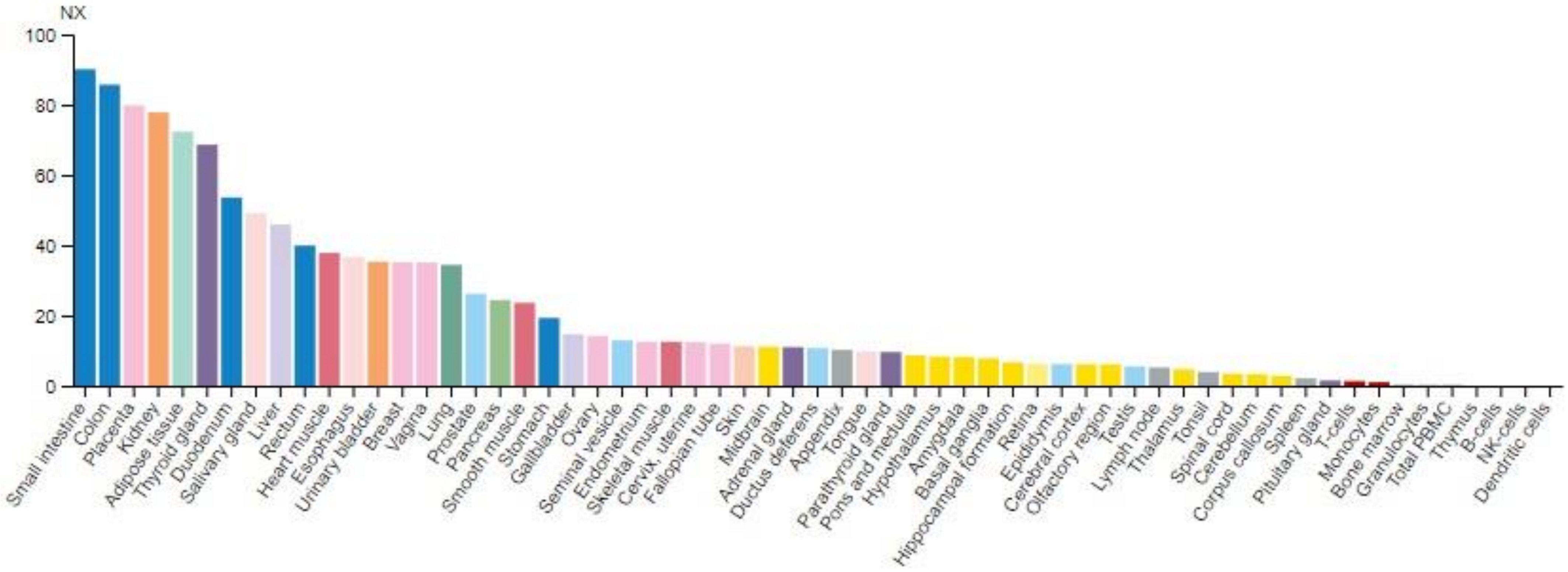

NX
100
80
60
40
20
0
Small intestine
Colon
Placenta
Kidney
Adipose tissue
Thyroid gland
Duodenum
Salivary gland
Liver
Rectum
Heart muscle
Esophagus
Urinary bladder
Breast
Vagina
Lung
Prostate
Pancreas
Smooth muscle
Stomach
Gallbladder
Ovary
Seminal vesicle
Endometrium
Skeletal muscle
Cervix, uterine
Fallopian tube
Skin
Midbrain
Adrenal gland
Ductus deferens
Appendix
Tongue
Parathyroid gland
Pons and medulla
Hypothalamus
Amygdala
Basal ganglia
Hippocampal formation
Retina
Epididymis
Cerebral cortex
Olfactory region
Testis
Lymph node
Thalamus
Tonsil
Spinal cord
Cerebellum
Corpus callosum
Spleen
Pituitary gland
T-cells
Monocytes
Bone marrow
Granulocytes
Total PBMC
Thymus
B-cells
NK-cells
Dendritic cells

SLC6A19

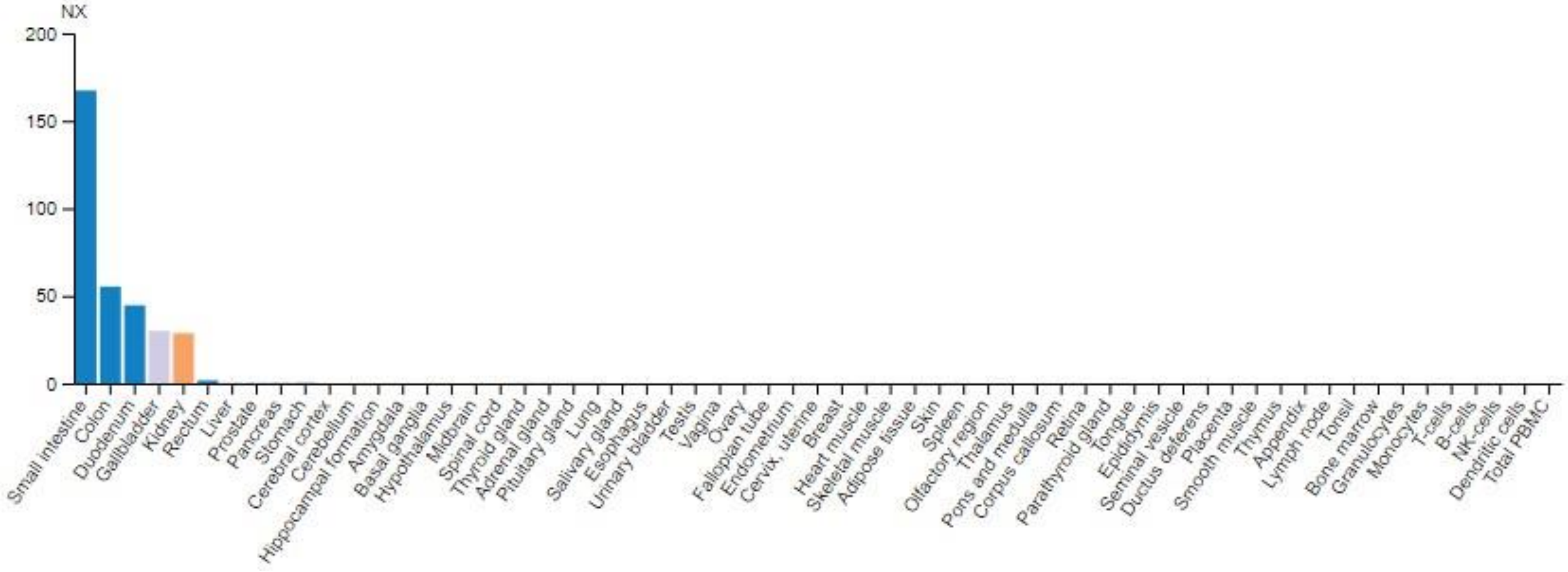

NX
200
150
100
50
0
Small intestine
Colon
Duodenum
Gallbladder
Kidney
Rectum
Liver
Prostate
Pancreas
Stomach
Cerebral cortex
Cerebellum
Hippocampal formation
Amygdala
Basal ganglia
Hypothalamus
Midbrain
Spinal cord
Thyroid gland
Adrenal gland
Pituitary gland
Lung
Salivary gland
Esophagus
Urinary bladder
Testis
Vagina
Ovary
Fallopian tube
Endometrium
Cervix, uterine
Breast
Heart muscle
Skeletal muscle
Adipose tissue
Skin
Spleen
Olfactory region
Thalamus
Pons and medulla
Corpus callosum
Retina
Parathyroid gland
Tongue
Epididymis
Seminal vesicle
Ductus deferens
Placenta
Smooth muscle
Thymus
Appendix
Lymph node
Tonsil
Bone marrow
Granulocytes
Monocytes
T-cells
B-cells
NK-cells
Dendritic cells
Total PBMC

SUT1A2

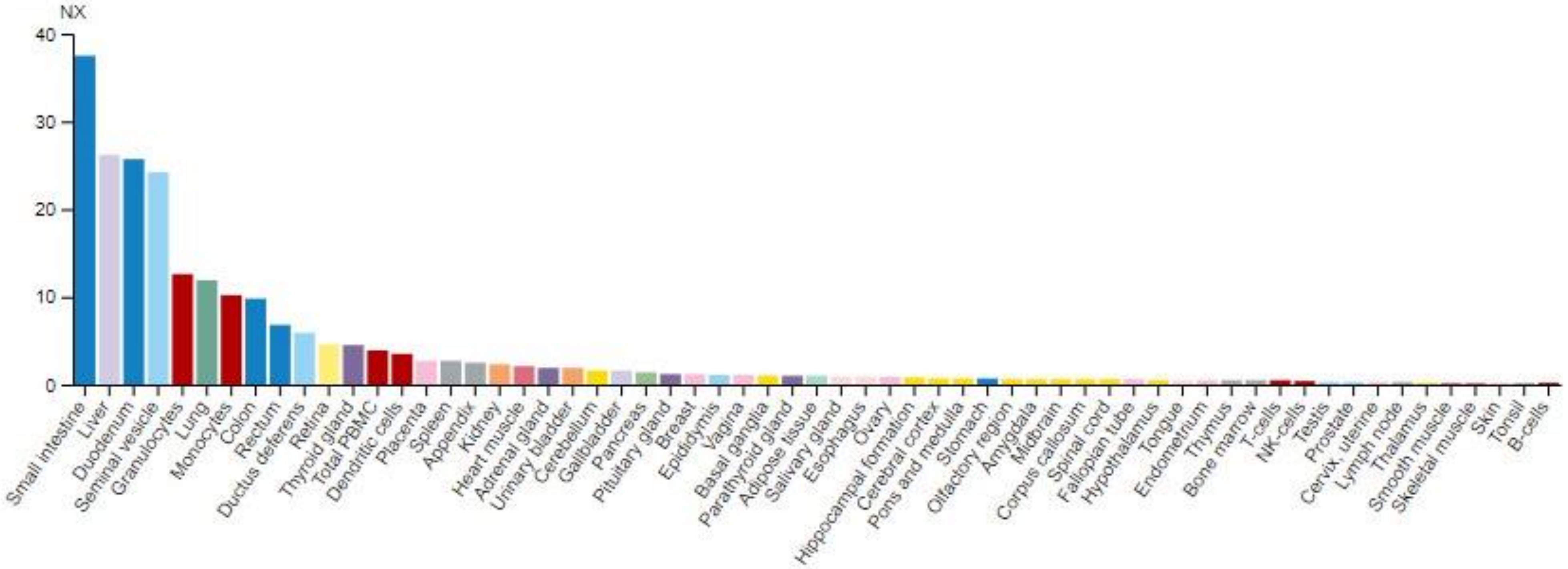
NX
40
30
20
10
0
Small intestine
Liver
Duodenum
Seminal vesicle
Granulocytes
Lung
Monocytes
Colon
Rectum
Ductus deferens
Retina
Thyroid gland
Total PBMC
Dendritic cells
Placenta
Spleen
Appendix
Kidney
Heart muscle
Adrenal gland
Urinary bladder
Cerebellum
Gallbladder
Pancreas
Pituitary gland
Breast
Epididymis
Vagina
Basal ganglia
Parathyroid gland
Adipose tissue
Salivary gland
Esophagus
Ovary
Hippocampal formation
Cerebral cortex
Pons and medulla
Stomach
Olfactory region
Amygdala
Midbrain
Corpus callosum
Spinal cord
Fallopian tube
Hypothalamus
Tongue
Endometrium
Thymus
Bone marrow
T-cells
NK-cells
Testis
Prostate
Cervix, uterine
Lymph node
Thalamus
Smooth muscle
Skeletal muscle
Skin
Tonsil
B-cells

CYP2D6

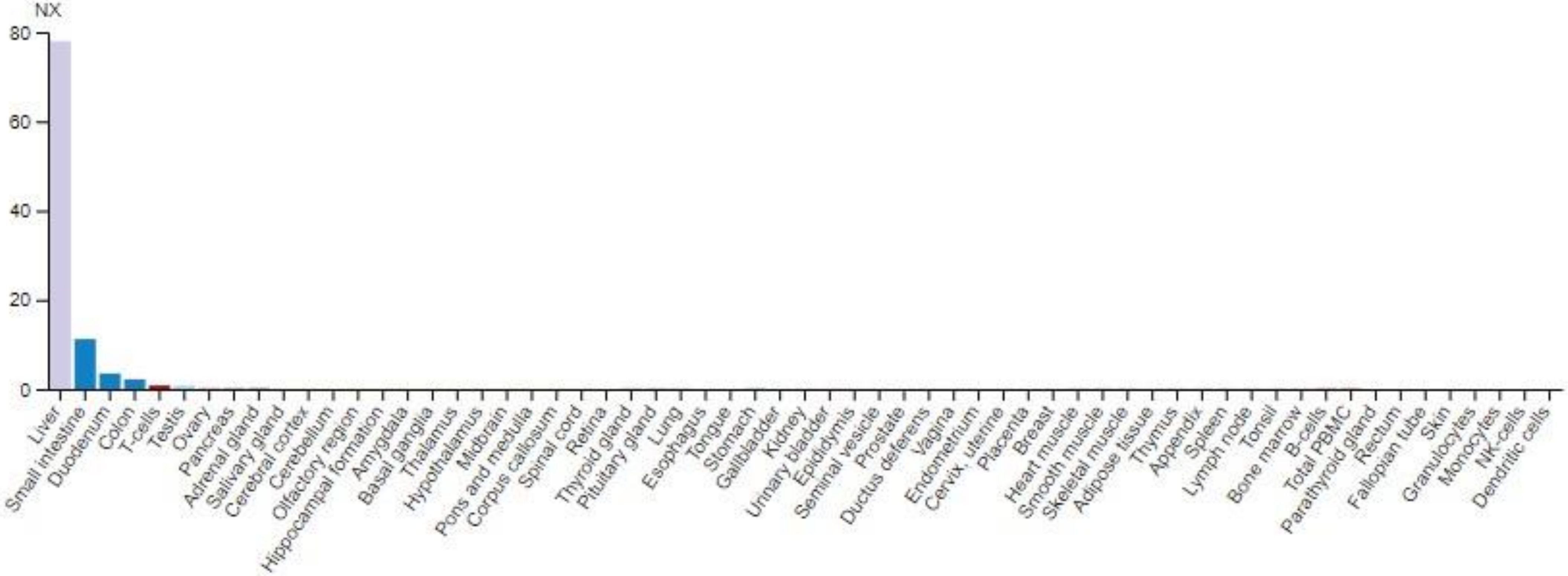
NX
80
60
40
20
0
Liver
Small intestine
Duodenum
Colon
T-cells
Testis
Ovary
Pancreas
Adrenal gland
Salivary gland
Cerebral cortex
Cerebellum
Olfactory region
Hippocampal formation
Amygdala
Basal ganglia
Thalamus
Hypothalamus
Midbrain
Pons and medulla
Corpus callosum
Spinal cord
Retina
Thyroid gland
Pituitary gland
Lung
Esophagus
Tongue
Stomach
Gallbladder
Kidney
Urinary bladder
Epididymis
Seminal vesicle
Prostate
Ductus deferens
Vagina
Endometrium
Cervix, uterine
Placenta
Breast
Heart muscle
Smooth muscle
Skeletal muscle
Adipose tissue
Thymus
Appendix
Spleen
Lymph node
Tonsil
Bone marrow
B-cells
Total PBMC
Parathyroid gland
Rectum
Fallopian tube
Skin
Granulocytes
Monocytes
NK-cells
Dendritic cells

SLC3A1

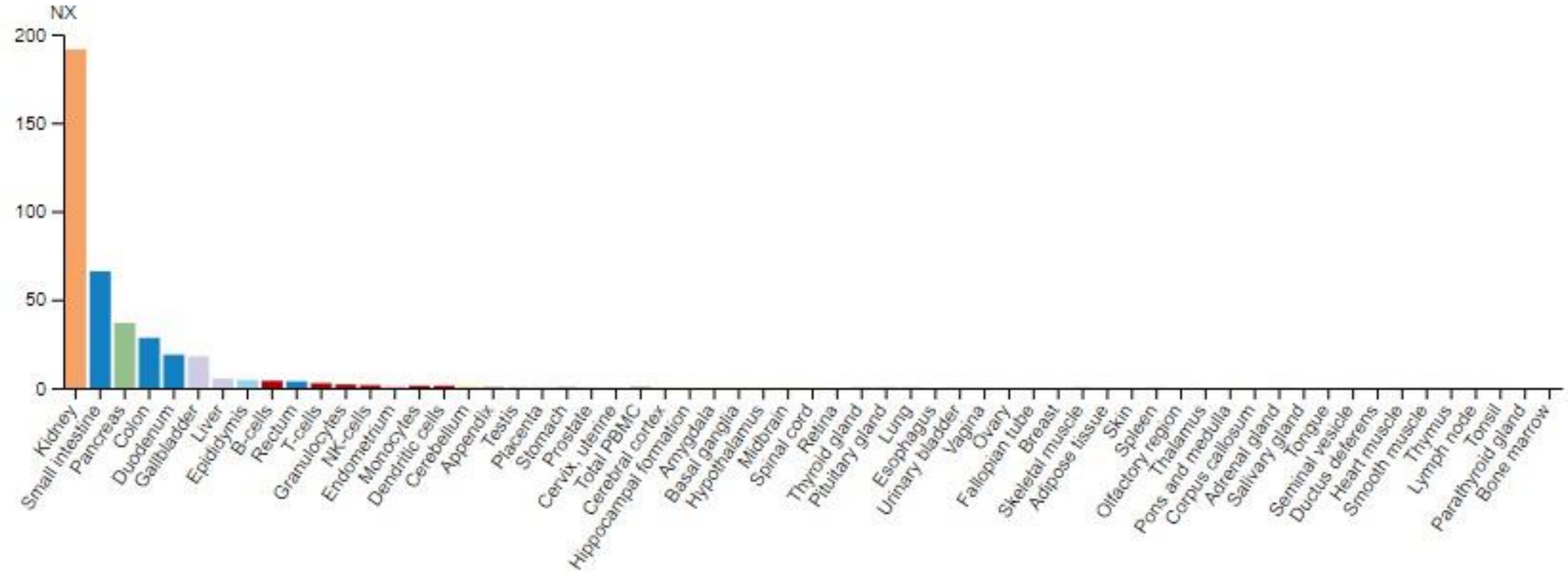
NX
200
150
100
50
0
Kidney
Small intestine
Pancreas
Colon
Duodenum
Gallbladder
Liver
Epididymis
B-cells
Rectum
T-cells
Granulocytes
NK-cells
Endometrium
Monocytes
Dendritic cells
Cerebellum
Appendix
Testis
Placenta
Stomach
Prostate
Cervix, uterine
Total PBMC
Cerebral cortex
Hippocampal formation
Amygdala
Basal ganglia
Hypothalamus
Midbrain
Spinal cord
Retina
Thyroid gland
Pituitary gland
Lung
Esophagus
Urinary bladder
Vagina
Ovary
Fallopian tube
Breast
Skeletal muscle
Adipose tissue
Skin
Spleen
Olfactory region
Thalamus
Pons and medulla
Corpus callosum
Adrenal gland
Salivary gland
Tongue
Seminal vesicle
Ductus deferens
Heart muscle
Smooth muscle
Thymus
Lymph node
Tonsil
Parathyroid gland
Bone marrow

SULT1A3

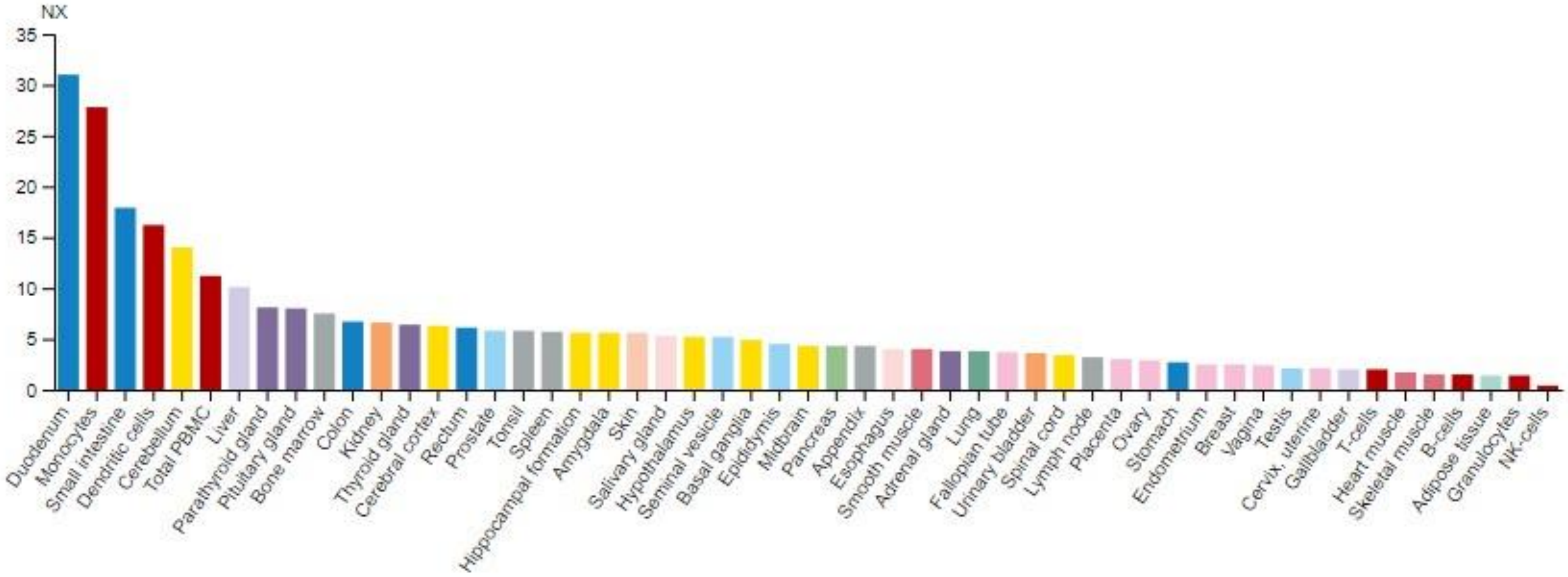
NX
35
30
25
20
15
10
5
0
Duodenum
Monocytes
Small intestine
Dendritic cells
Cerebellum
Total PBMC
Liver
Parathyroid gland
Pituitary gland
Bone marrow
Colon
Kidney
Thyroid gland
Cerebral cortex
Rectum
Prostate
Tonsil
Spleen
Hippocampal formation
Amygdala
Skin
Salivary gland
Hypothalamus
Seminal vesicle
Basal ganglia
Epididymis
Midbrain
Pancreas
Appendix
Esophagus
Smooth muscle
Adrenal gland
Lung
Fallopian tube
Urinary bladder
Spinal cord
Lymph node
Placenta
Ovary
Stomach
Endometrium
Breast
Vagina
Testis
Cervix, uterine
Gallbladder
T-cells
Heart muscle
Skeletal muscle
B-cells
Adipose tissue
Granulocytes
NK-cells

SUT1A1

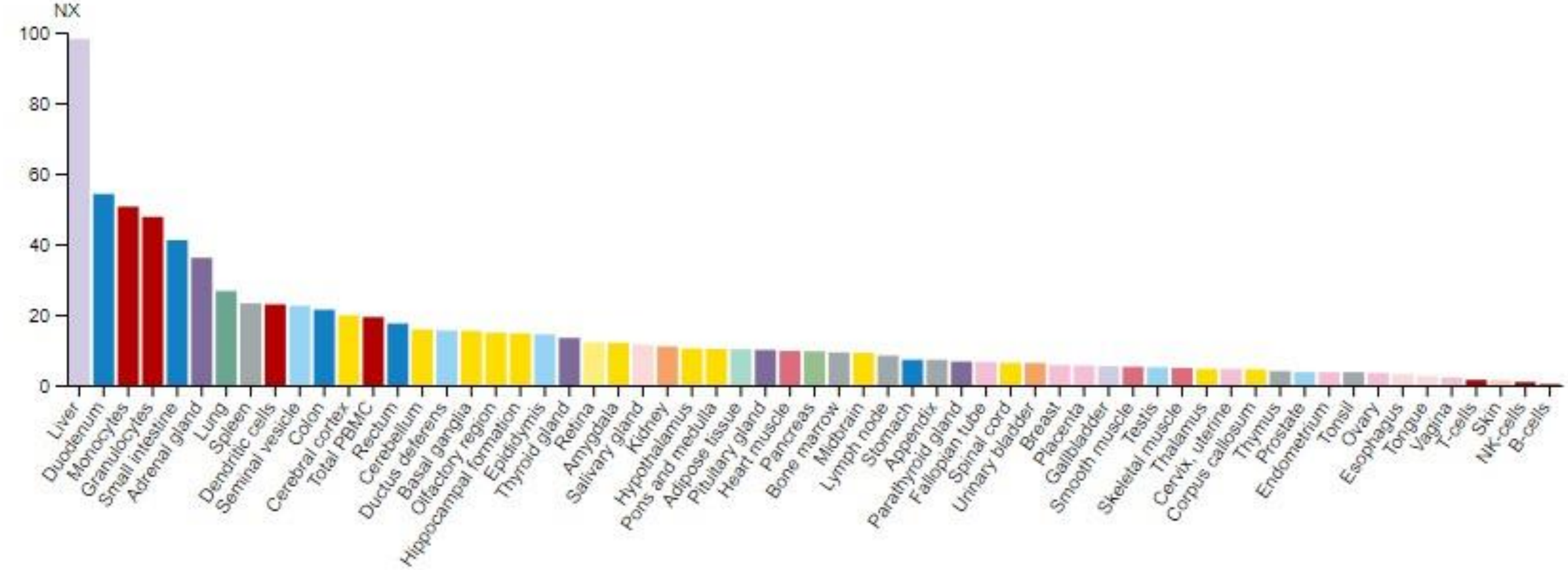
NX
100
80
60
40
20
0
Liver
Duodenum
Monocytes
Granulocytes
Small intestine
Adrenal gland
Lung
Spleen
Dendritic cells
Seminal vesicle
Colon
Cerebral cortex
Total PBMC
Rectum
Cerebellum
Ductus deferens
Basal ganglia
Olfactory region
Hippocampal formation
Epididymis
Thyroid gland
Retina
Amygdala
Salivary gland
Kidney
Hypothalamus
Pons and medulla
Adipose tissue
Pituitary gland
Heart muscle
Pancreas
Bone marrow
Midbrain
Lymph node
Stomach
Appendix
Parathyroid gland
Fallopian tube
Spinal cord
Urinary bladder
Breast
Placenta
Gallbladder
Smooth muscle
Testis
Skeletal muscle
Thalamus
Cervix, uterine
Corpus callosum
Thymus
Prostate
Endometrium
Tonsil
Ovary
Esophagus
Tongue
Vagina
T-cells
Skin
NK-cells
B-cells

SLC16A10

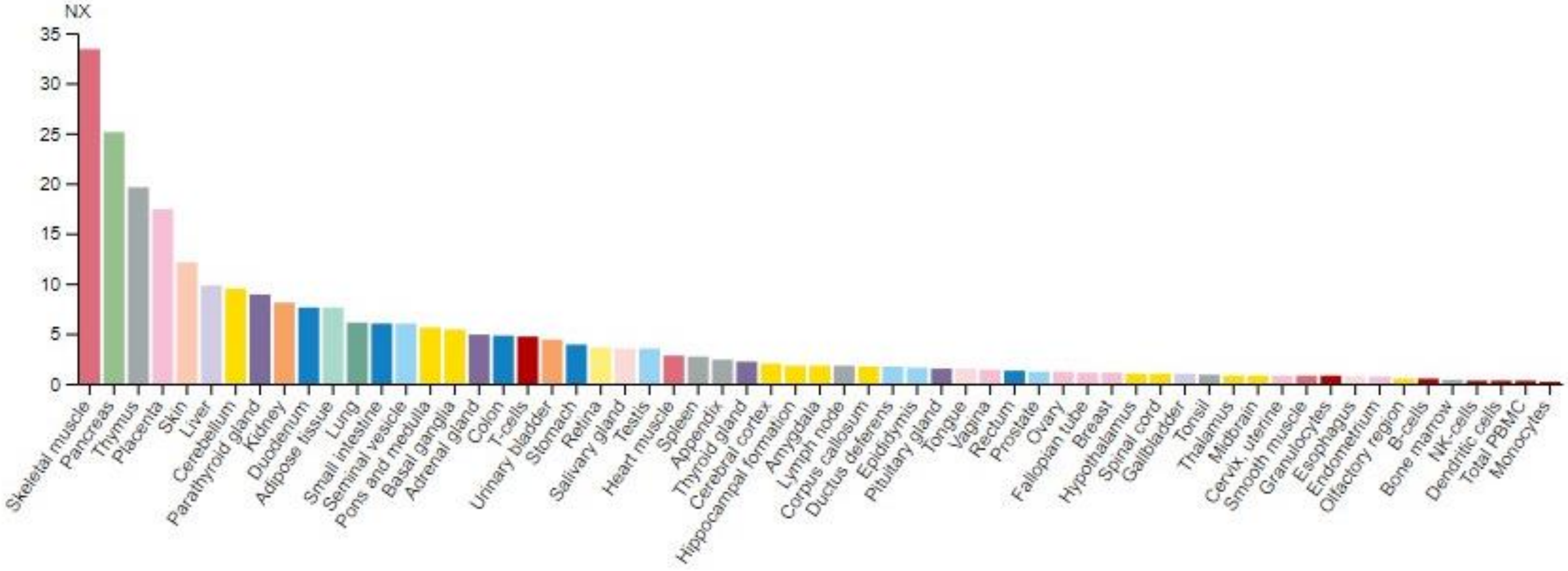

NX
35
30
25
20
15
10
5
0
Skeletal muscle
Pancreas
Thymus
Placenta
Skin
Liver
Cerebellum
Parathyroid gland
Kidney
Duodenum
Adipose tissue
Lung
Small intestine
Seminal vesicle
Pons and medulla
Basal ganglia
Adrenal gland
Colon
T-cells
Urinary bladder
Stomach
Retina
Salivary gland
Testis
Heart muscle
Spleen
Appendix
Thyroid gland
Cerebral cortex
Hippocampal formation
Amygdala
Lymph node
Corpus callosum
Ductus deferens
Epididymis
Pituitary gland
Tongue
Vagina
Rectum
Prostate
Ovary
Fallopian tube
Breast
Hypothalamus
Spinal cord
Gallbladder
Tonsil
Thalamus
Midbrain
Cervix, uterine
Smooth muscle
Granulocytes
Esophagus
Endometrium
Olfactory region
B-cells
Bone marrow
NK-cells
Dendritic cells
Total PBMC
Monocytes

MAOB

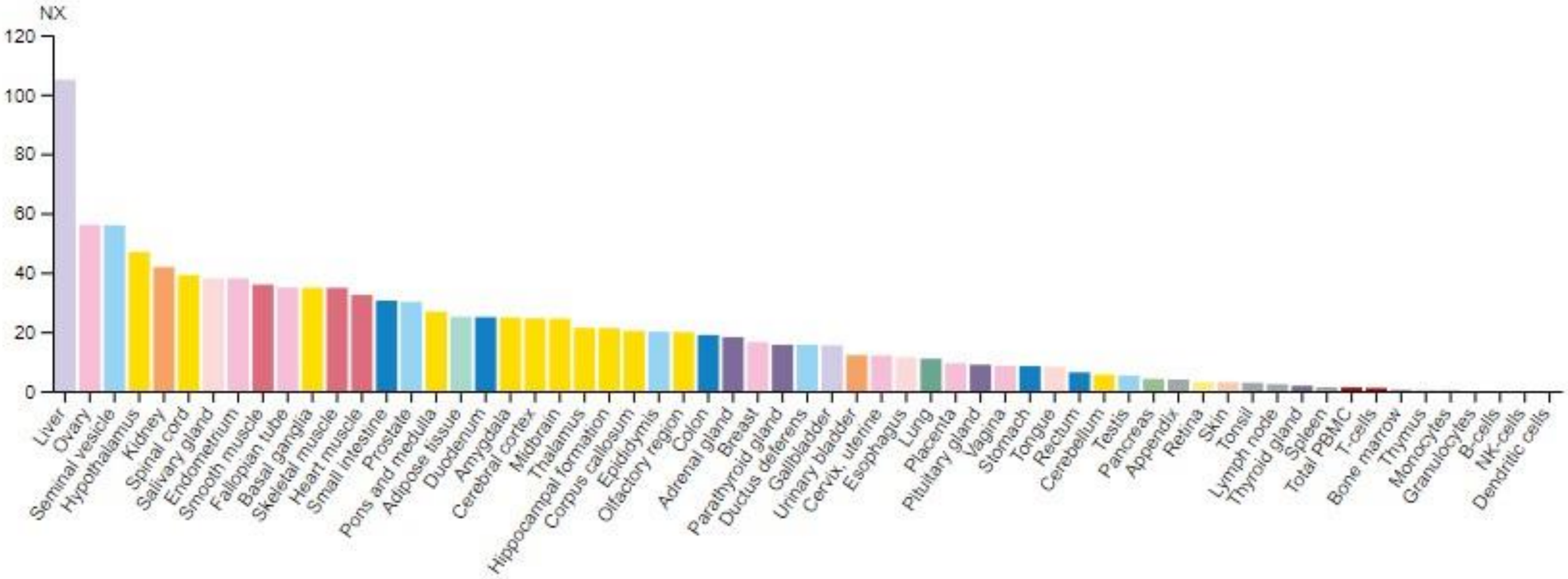

NX
120
100
80
60
40
20
0
Liver
Ovary
Seminal vesicle
Hypothalamus
Kidney
Spinal cord
Salivary gland
Endometrium
Smooth muscle
Fallopian tube
Basal ganglia
Skeletal muscle
Heart muscle
Small intestine
Prostate
Pons and medulla
Adipose tissue
Duodenum
Amygdala
Cerebral cortex
Midbrain
Thalamus
Hippocampal formation
Corpus callosum
Epididymis
Olfactory region
Colon
Adrenal gland
Breast
Parathyroid gland
Ductus deferens
Gallbladder
Urinary bladder
Cervix, uterine
Esophagus
Lung
Placenta
Pituitary gland
Vagina
Stomach
Tongue
Rectum
Cerebellum
Testis
Pancreas
Appendix
Retina
Skin
Tonsil
Lymph node
Thyroid gland
Spleen
Total PBMC
T-cells
Bone marrow
Thymus
Monocytes
Granulocytes
B-cells
NK-cells
Dendritic cells

SLC3A2

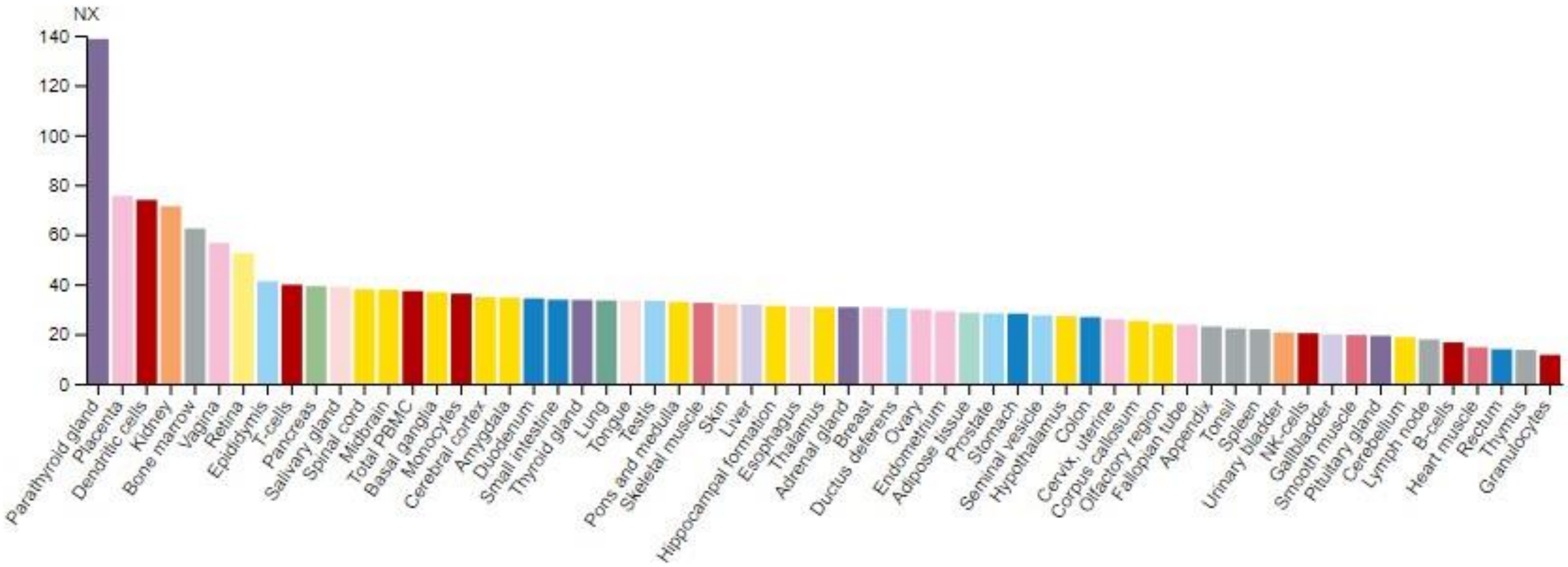
NX
140
120
100
80
60
40
20
0
Parathyroid gland
Placenta
Dendritic cells
Kidney
Bone marrow
Vagina
Retina
Epididymis
T-cells
Pancreas
Salivary gland
Spinal cord
Midbrain
Total PBMC
Basal ganglia
Monocytes
Cerebral cortex
Amygdala
Duodenum
Small intestine
Thyroid gland
Lung
Tongue
Testis
Pons and medulla
Skeletal muscle
Skin
Liver
Hippocampal formation
Esophagus
Thalamus
Adrenal gland
Breast
Ductus deferens
Ovary
Endometrium
Adipose tissue
Prostate
Stomach
Seminal vesicle
Hypothalamus
Colon
Cervix, uterine
Corpus callosum
Olfactory region
Fallopian tube
Appendix
Tonsil
Spleen
Urinary bladder
NK-cells
Gallbladder
Smooth muscle
Pituitary gland
Cerebellum
Lymph node
B-cells
Heart muscle
Rectum
Thymus
Granulocytes

SLC7A8

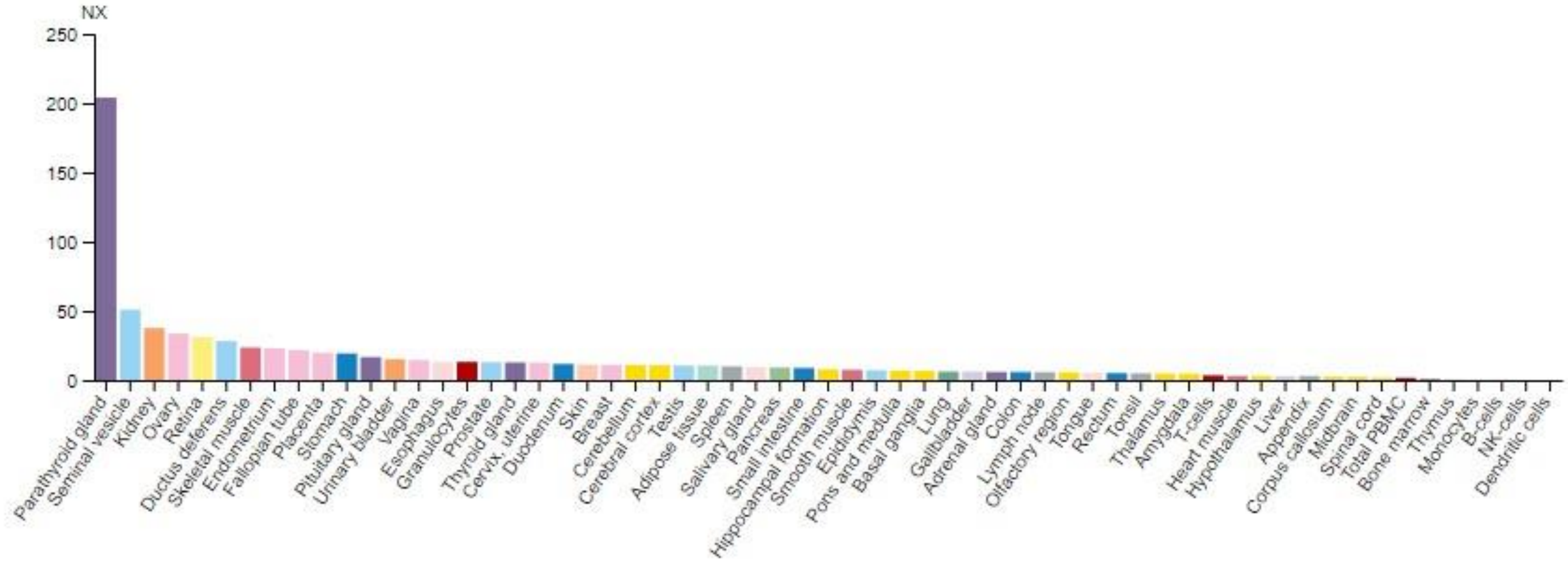
NX
250
200
150
100
50
0
Parathyroid gland
Seminal vesicle
Kidney
Ovary
Retina
Ductus deferens
Skeletal muscle
Endometrium
Fallopian tube
Placenta
Stomach
Pituitary gland
Urinary bladder
Vagina
Esophagus
Granulocytes
Prostate
Thyroid gland
Cervix, uterine
Duodenum
Skin
Breast
Cerebellum
Cerebral cortex
Testis
Adipose tissue
Spleen
Salivary gland
Pancreas
Small intestine
Hippocampal formation
Smooth muscle
Epididymis
Pons and medulla
Basal ganglia
Lung
Gallbladder
Adrenal gland
Colon
Lymph node
Olfactory region
Tongue
Rectum
Tonsil
Thalamus
Amygdala
T-cells
Heart muscle
Hypothalamus
Liver
Appendix
Corpus callosum
Midbrain
Spinal cord
Total PBMC
Bone marrow
Thymus
Monocytes
B-cells
NK-cells
Dendritic cells

TH

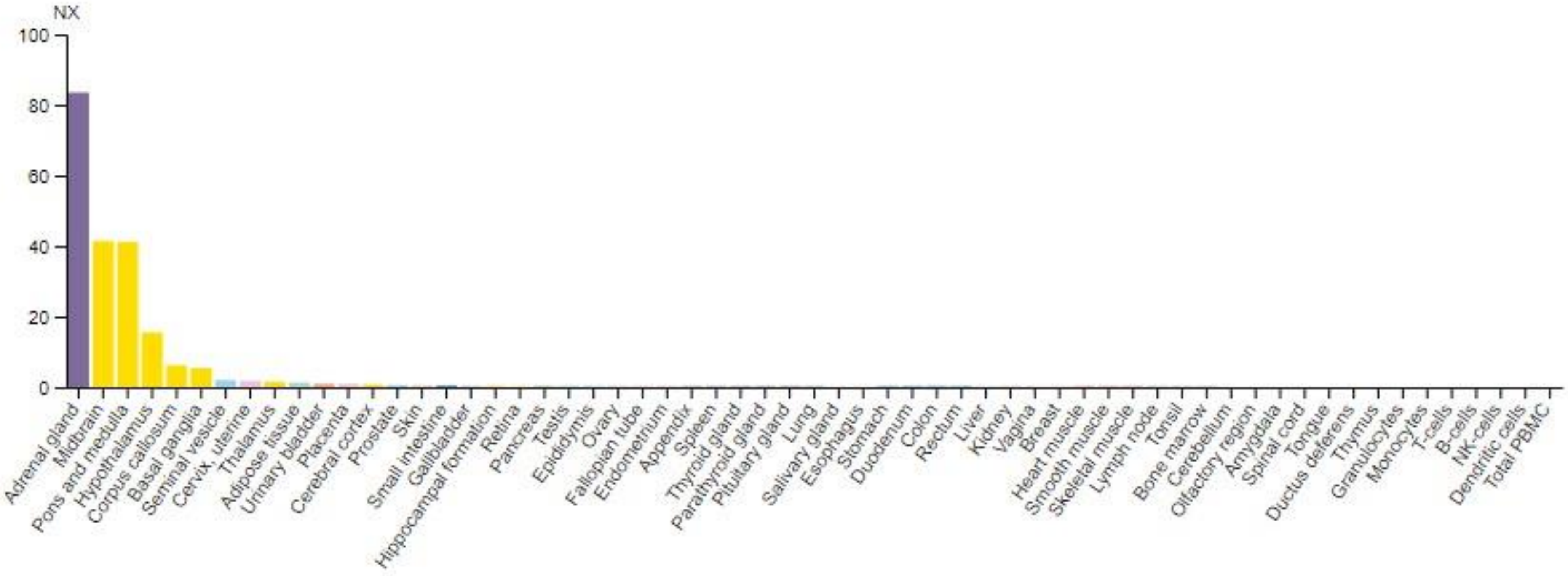
NX
100
80
60
40
20
0
Adrenal gland
Midbrain
Pons and medulla
Hypothalamus
Corpus callosum
Basal ganglia
Seminal vesicle
Cervix, uterine
Thalamus
Adipose tissue
Urinary bladder
Placenta
Cerebral cortex
Prostate
Skin
Small intestine
Gallbladder
Hippocampal formation
Retina
Pancreas
Testis
Epididymis
Ovary
Fallopian tube
Endometrium
Appendix
Spleen
Thyroid gland
Parathyroid gland
Pituitary gland
Lung
Salivary gland
Esophagus
Stomach
Duodenum
Colon
Rectum
Liver
Kidney
Vagina
Breast
Heart muscle
Smooth muscle
Skeletal muscle
Lymph node
Tonsil
Bone marrow
Cerebellum
Olfactory region
Amygdala
Spinal cord
Tongue
Ductus deferens
Thymus
Granulocytes
Monocytes
T-cells
B-cells
NK-cells
Dendritic cells
Total PBMC
