# Supplementary material for: Molecular Insights into SARS-CoV2-Induced Alterations of the Gut/Brain Axis
Source: Int J Mol Sci. 2021 Sep 28;22(19):10440. doi: 10.3390/ijms221910440 (PMC8508788; doi:10.3390/ijms221910440)
Supplement: Supplementary file 1 [file ijms-22-10440-s001.zip › Table S1.pdf]

Table S1: List of the top-25 genes that more closely co-express with *ACE2* in SARS-CoV2-infected human enterocytes. A set of previously published RNA-seq data [34] obtained from the analysis of SARS-CoV2-infected human intestinal organoids was re-assessed in order to identify the top-25 genes that more closely co-express with *ACE2* under these experimental conditions.

Gene Symbol	Gene Name
ACSL5	acyl-CoA synthetase long-chain family member 5
ALDOB	aldolase B, fructose-bisphosphate
ATP1A1	ATPase, Na+/K+ transporting, alpha 1 polypeptide
BTD	biotinidase
CENPJ	centromere protein J
CREB3L3	cAMP responsive element binding protein 3-like 3
CYP3A4	cytochrome P450, family 3, subfamily A, polypeptide 4
DMD	dystrophin
GATM	glycine amidinotransferase
GLRX	glutaredoxin
GSTM4	glutathione S-transferase mu 4
IGIP	IgA-inducing protein homolog
MAOB	monoamine oxidase B
MTTP	microsomal triglyceride transfer protein
OAT	ornithine aminotransferase
PCYOX1	prenylcysteine oxidase 1
PDCD4	programmed cell death 4
PHGR1	proline/histidine/glycine-rich 1
PLD1	phospholipase D1
RARRES1	retinoic acid receptor responder
RBP2	retinol binding protein 2
SGK1	serum/glucocorticoid regulated kinase 1
SLC5A1	solute carrier family 5
TMBIM6	transmembrane BAX inhibitor motif containing 6
TTC36	tetratricopeptide repeat domain 36
