# Supplementary material for: Molecular Insights into SARS-CoV2-Induced Alterations of the Gut/Brain Axis
Source: Int J Mol Sci. 2021 Sep 28;22(19):10440. doi: 10.3390/ijms221910440 (PMC8508788; doi:10.3390/ijms221910440)
Supplement: Supplementary file 1 [file ijms-22-10440-s001.zip › Table S2.pdf]

Table S2: Enrichment analysis of the *ACE2* co-expression network. An enrichment analysis was performed on the list of top-25 genes that that more closely co-express with *ACE2* in SARS-CoV2-infected human enterocytes. To this aim we used the Enrichr pletform of enrichment analyses (https://maayanlab.cloud/Enrichr/) and queried the BioPlanet library of pathways (created and run by the National Institute of Health, USA) [37], which compiles and integrate libraries of pathways from a large range of sources. The top-10 most significantly enriched pathways, the corresponding adjusted p-values and genes annotated with each pathway are shown below.

**Pathway**	**Adjusted P-value**	**Genes**
Metabolism	1.78E-4	*GSTM4, GATM, OAT, BTD, MAOB, MTTP, ACSL5, GLRX, ALDOB, CYP3A4, PLD1*
Arginine and proline metabolism	0.002	*GATM, OAT, MAOB*
Drug metabolism: cytochrome P450	0.006	*GSTM4, MAOB, CYP3A4*
Urea cycle and metabolism of amino groups	0.008	*GATM, OAT*
mTOR signaling pathway	0.009	*PDCD4, PLD1, SGK1*
Biological oxidations	0.011	*GSTM4, MAOB, CYP3A4*
Glycine, serine and threonine metabolism	0.011	*GATM, MAOB*
Alpha-synuclein signaling	0.011	*MAOB, PLD1*
Aldosterone-regulated sodium reabsorption	0.017	*ATP1A1, SGK1*
Tryptophan metabolism	0.029	*MAOB, CYP3A4*
