# Supplementary material for: Molecular Insights into SARS-CoV2-Induced Alterations of the Gut/Brain Axis
Source: Int J Mol Sci. 2021 Sep 28;22(19):10440. doi: 10.3390/ijms221910440 (PMC8508788; doi:10.3390/ijms221910440)
Supplement: Supplementary file 1 [file ijms-22-10440-s001.zip › Table S3.pdf]

Table S3: Extracted results showing statistically significant fold changes in blood levels of relevant metabolites in comparisons of non severe COVID-19 patients vs healthy subjects (1st table) and severe COVID-19 patients vs healthy subjects (2nd table) [72].

Non Severe COVID-19 vs Healthy subjects		
**Metabolites**	**Fold change**	**Adjusted p-value**
asparagine	-0,675798	7,5216E-07
glutamine	-0,294186	0,000990825
glycine	-0,571081	8,06036E-06
isoleucine	-0,613711	1,11056E-05
leucine	-0,510385	5,67243E-06
methionine	-0,713963	3,19159E-08
proline	-0,870753	6,94713E-08
serine	-0,435634	0,000117601
threonine	-0,80806	5,31282E-07
tryptophan	-0,639467	2,40902E-07
tyrosine	-0,323365	0,034459511
valine	-0,550451	1,69336E-07
dopamine 3-O-sulfate	-0,998589	0,001989449
**Severe COVID-19 vs Healthy subjects**		
glutamine	-0,316643	0,014151896
glycine	-0,580321	0,000207618
isoleucine	-0,79801	1,90408E-05
leucine	-0,533004	0,000523773
methionine	-0,583045	0,000290771
proline	-0,923271	2,51193E-07
serine	-0,409389	0,002062248
threonine	-0,785386	3,41246E-05
tryptophan	-0,567395	0,000284531
valine	-0,548777	0,000206703
